# Genomic Approaches Reveal an Endemic Subpopulation of Gray Wolves in Southern China

**DOI:** 10.1016/j.isci.2019.09.008

**Published:** 2019-09-10

**Authors:** Guo-Dong Wang, Ming Zhang, Xuan Wang, Melinda A. Yang, Peng Cao, Feng Liu, Heng Lu, Xiaotian Feng, Pontus Skoglund, Lu Wang, Qiaomei Fu, Ya-Ping Zhang

**Affiliations:** 1 State Key Laboratory of Genetic Resources and Evolution, Kunming Institute of Zoology, Chinese Academy of Sciences, Kunming 650223, China; 2 Center for Excellence in Animal Evolution and Genetics, Chinese Academy of Sciences, Kunming 650223, China; 3 Key Laboratory of Vertebrate Evolution and Human Origins of Chinese Academy of Sciences, IVPP, CAS, Beijing 100044, China; 4 Center for Excellence in Life and Paleoenvironment, Chinese Academy of Sciences, Beijing 100044, China; 5 University of Chinese Academy of Sciences, Beijing 100049, China; 6 Department of Molecular and Cell Biology, School of Life Sciences, University of Science and Technology of China, Hefei 230026, China; 7 The Francis Crick Institute, London NW1 1AT, UK; 8 State Key Laboratory for Conservation and Utilization of Bio-Resources in Yunnan, Yunnan University, Kunming 650091, China

**Keywords:** Biological Sciences, Genomics, Evolutionary Biology

## Abstract

Although gray wolves (*Canis lupus*) are one of the most widely distributed terrestrial mammals, their origins in China are not well understood. We sequenced six specimens from wolf skins, showing that gray wolves from Southern China (SC) derive from a single lineage, distinct from gray wolves from the Tibetan Plateau and Northern China, suggesting that SC gray wolves may form a distinct subpopulation. Of SC gray wolves, one wolf from Zhejiang carries a genetic component from a canid and had gene flow from a population related to or further diverged from wolves than the dhole. This may indicate that interspecific gene flow likely played an important role in shaping the speciation patterns and population structure in the genus *Canis*. Our study is the first to survey museum gray wolves' genomes from Southern China, highlighting how sequencing the paleogenome from museum specimens can help us to study extinct species.

## Highlights

• Gray wolves from South China derive from a single lineage
• A Zhejiang wolf shows admixture (∼12%–14%) from a deeply diverged canid species
• Lowland gray wolf shows close genetic relationship to Tibetan Plateau gray wolves

## Graphical Abstract

**Figure:**
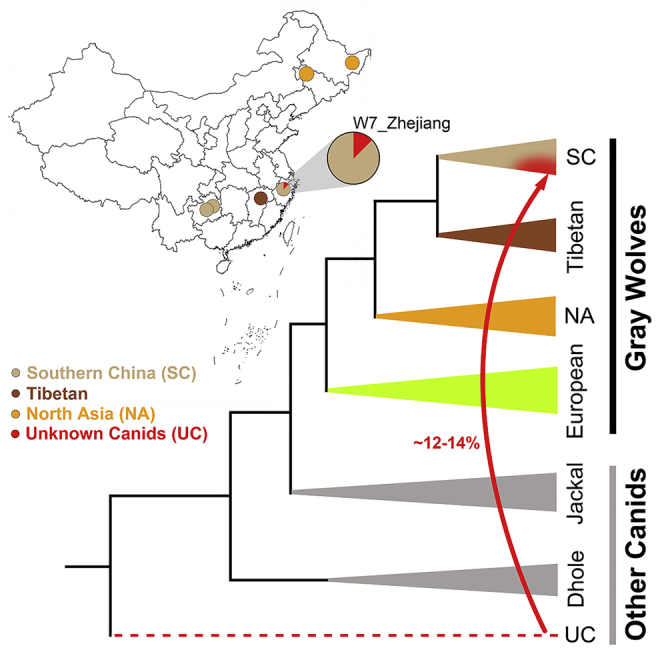

## Introduction

The place of origin for domestic dogs (*Canis lupus familiaris*) remains a controversial question for the scientific community despite many efforts at studying dog domestication (Botigué et al., 2017, Frantz et al., 2016, Liu et al., 2018, Shannon et al., 2015, Thalmann et al., 2013, Vonholdt et al., 2010, Wang et al., 2016a). Geographic distribution, population structure, and genomic features of wild ancestors are essential factors to determine sources of domestication (Wang et al., 2016b). Gray wolves (*Canis lupus*) are the closest wild relative of dogs, and they are also one of the most widely distributed terrestrial mammals, originally inhabiting major parts of Eurasia, North America, and North Africa (Loog et al., 2018, Wilson and Reeder, 2005, Young et al., 1944). Previous studies suggested that gray wolves have a complex history (vonHoldt et al., 2011, Wayne et al., 1992), with subpopulation structure related to local niches (Carmichael et al., 2001, Geffen et al., 2004, Pilot et al., 2006, Pilot et al., 2014) and long-term genetic admixture not only with dogs (Freedman et al., 2014, Liu et al., 2018) but also with coyotes (Fan et al., 2016, Monzon et al., 2014, Pilot et al., 2014, vonHoldt et al., 2016, vonHoldt et al., 2011). In China, gray wolves were distributed across the mainland, including most southern regions (Smith and Xie, 2014, Wang et al., 2016b). Genomic approaches using gray wolf specimens from Southern China (SC) may help to shed new light on the demographic history of gray wolves and domestic dogs.

## Results and Discussion

**Figure 1:**
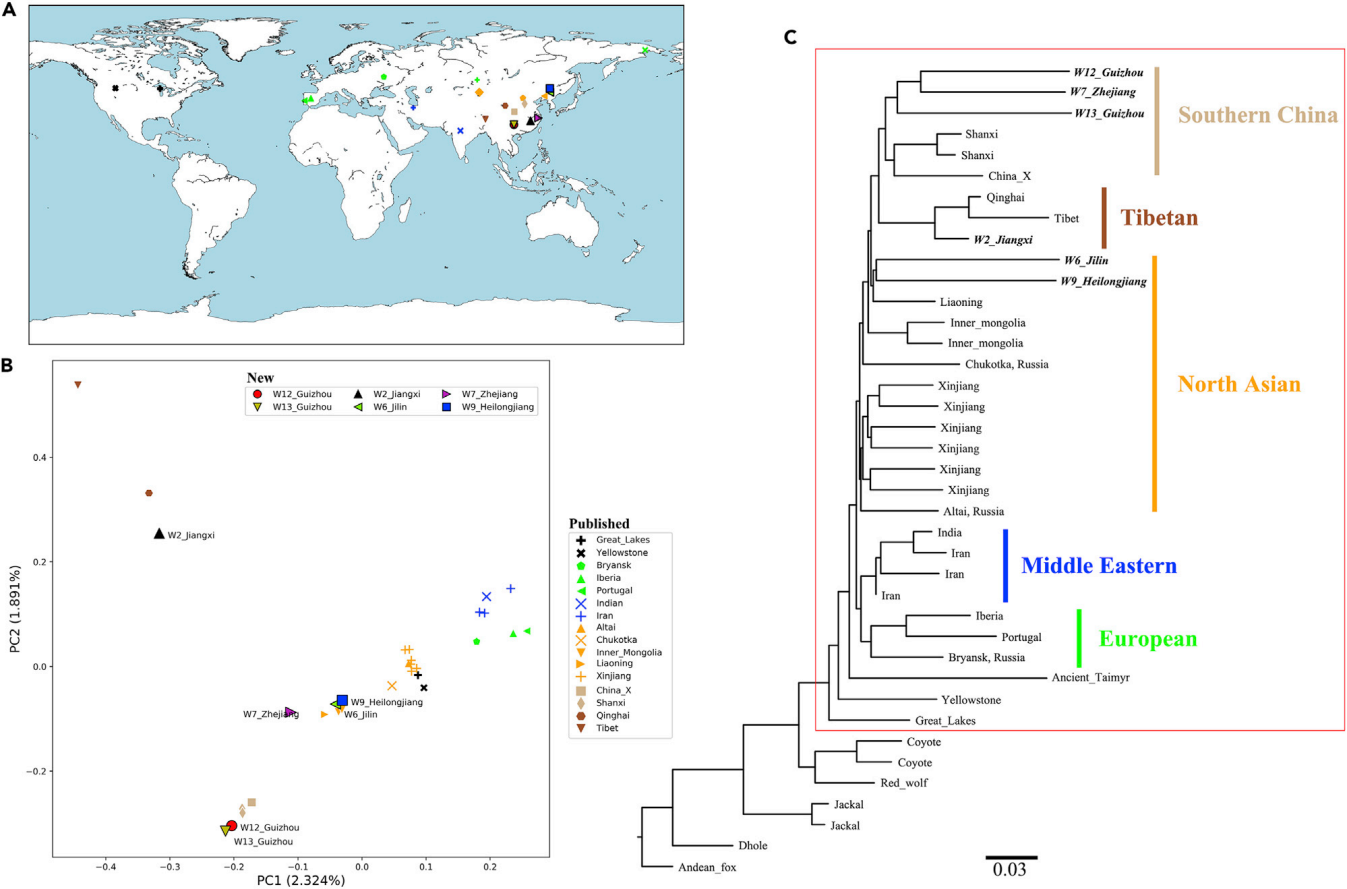
Geographic Locations, Population Structure of 31 Gray Wolves, and Phylogeny of 39 Canids (A and B) (A) Geographic locations, where the key is shared with the principal-component analysis in (B). In (B), the label “New” represents the six samples sequenced in the study, and the label “Published” represents 25 samples from previous studies. (C) The maximum likelihood tree of 39 canids, where the Andean fox is used as an outgroup. All the gray wolves are in the red line, and the newly sequenced individuals are marked in bold and italics.

From two natural history museums in China, we obtained six historical gray wolf skin samples collected from Mainland China (Figures 1A and S1 and Table 1, detailed description in Wang et al., 2016b). More details for the samples are shown in the methods (Transparent Methods). As skin samples were treated with chemical reagents and underwent special processing for preservation during storage and exhibition in museums, we used a modified ancient DNA (aDNA) protocol (Dabney et al., 2013) to retrieve genetic material from the skin samples. In total, 35 genomic libraries were produced for these six samples using a double-stranded library preparation protocol (Kircher et al., 2012, Meyer and Kircher, 2010), and each was treated with uracil-DNA glycosylase and endonuclease (Endo VIII) to remove characteristic aDNA deamination (Briggs et al., 2007) (Table S1). We sequenced the libraries using 2×150-bp reads on an Illumina HiSeq X platform.

**Table 1:** Information on Samples Sequenced in This Study

Sample ID	Coverage	SNPs	Sources	Location
W6_Jilin	1.87	6,890,236	National Zoological Museum of China, Beijing, China	Jilin
W7_Zhejiang	0.15	1,866,574		Zhejiang
W9_Heilongjiang	1.61	5,438,606		Heilongjiang
W12_Guizhou	15.33	12,688,043	Kunming Natural History Museum of Zoology, Yunnan, China	Guizhou
W13_Guizhou	12.39	12,968,150		Guizhou
W2_Jiangxi	37.00	13,528,667		Jiangxi

We included 103 canids from previous studies (Auton et al., 2013, Botigué et al., 2017, Decker et al., 2015, Freedman et al., 2014, Marsden et al., 2016, Skoglund et al., 2015, Tang et al., 2019, vonHoldt et al., 2016, Wang et al., 2019a, Wang et al., 2016a, Wang et al., 2013, Wang et al., 2019b, Zhang et al., 2014), including an ancient gray wolf, Taimyr; 25 modern gray wolves chosen from regions overlapping their current ranges; 70 domestic dogs from all over the world; two jackals; two coyotes; one red wolf; one dhole; and one Andean fox (Figure 1, Table S2). We obtained 13.74 million SNPs, including 4.25 million transversions for further analysis (Transparent Methods).

All samples were sequenced to ∼0.15- to 15.3-fold average coverage except for the Jiangxi wolf (W2_Jiangxi), which was sequenced to 37.00-fold coverage (Table 1). Because most samples have low average coverage, we focused on SNPs previously found for modern canids (Marsden et al., 2016, Wang et al., 2016a), for which we called haploid alleles using randomly chosen sequence reads for every sample except W2_Jiangxi. We applied a filter where we ignored fragments with length less than 30, ignored the first and last two base pairs of each fragment, and required a base pair quality higher than 20 and mapping quality of at least 30. For the high-coverage W2_Jiangxi (Table 1) as well as the two medium-coverage Guizhou wolves (W12_Guizhou and W13_Guizhou), we called heterozygotes using the software GATK with the Unified Genotyper parameter to determine diploid calls (DePristo et al., 2011). Results using diploid calls for W12_Guizhou and W13_Guizhou are similar to results using random calls without heterozygous sites.

### Phylogeny and Population Structure

To investigate the relationship of the newly sampled individuals to wolf and dog populations, we calculated pairwise allele-sharing distances among all pairs of wolf and dog populations. We applied a principal-components analysis (PCA) to the resulting pairwise distance matrix using SMARTPCA (version: 13050) (Patterson et al., 2006). The first principal component distinguishes between gray wolf and dog populations, whereas the second principal component distinguishes between East Asian and European dogs (Figure S2), consistent with previous studies (Frantz et al., 2016, Vonholdt et al., 2010, Wang et al., 2016a). To obtain increased resolution, we redid the PCA excluding dog populations (Figure 1B). The resulting PCA shows that the two new Guizhou wolves (W12_Guizhou and W13_Guizhou) cluster with a Chinese wolf from the San Diego Zoo, whose origin is not recorded (labeled as “China_X” in this study) (Freedman et al., 2014), and two gray wolves from Shanxi, China, sampled in 1988 near the border of SC (Wang et al., 2016a). We also find that the new Jilin and Heilongjiang wolves (W6_Jilin and W9_Heilongjiang) cluster with gray wolves from Inner Mongolia and Liaoning. The new Jiangxi wolf (W2_Jiangxi) is closest to the Qinghai wolf, whereas the Zhejiang wolf (W7_Zhejiang) is closest to the cluster containing the gray wolves from Inner Mongolia and Liaoning (Figure S1).

We constructed a maximum likelihood (ML) tree (Figure 1C) and a neighbor-joining tree with only wolves (Figure S3), from which we further find that the Zhejiang wolf forms a clade with the Guizhou, Shanxi, and China_X wolves (Figure 1C). We define these gray wolves as the gray wolves from SC and find that they are most closely related to the Qinghai, Tibet, and Jiangxi wolves, where the Tibetan gray wolf (*Canis lupus chanco*) is a gray wolf subspecies that occupies habitats on the Qinghai-Tibet Plateau (Pocock, 1941) and possesses adaptations to high-altitude environments (Zhang et al., 2014). Other gray wolves from Northern Asia (NA, e.g., W6_Jilin, W9_Heilongjiang, and Liaoning) form a clade with the SC and Tibetan gray wolves relative to other NA, Middle Eastern, and European gray wolves that form a distinct clade with each other. The 35,000-year-old wolf from the Taimyr Peninsula in Northern Siberia joins at the base of the Eurasian wolf phylogeny, and American wolves separate the earliest from other wolves, consistent with Freedman et al. (2014).

We used TreeMix (v. 1.13) (Pickrell and Pritchard, 2012) to investigate the genetic relationship between historical and present-day wolves. TreeMix determines population structure using ML trees and allows for both population splits and potential gene flow by using genome-wide allele frequency data and a Gaussian approximation of genetic drift. The ML tree (Figure S4) without admixture (m = 0) is consistent with previous patterns, wherein gray wolves from East Asia form three groups: Guizhou and Zhejiang wolves form a clade with Shanxi and China_X wolves, the Jiangxi wolf forms a clade with Tibet and Qinghai wolves, and Jilin and Heilongjiang wolves, like other NA wolves, are outgroup to SC and Tibetan wolves. All three of these groups form a clade relative to non-Asian wolves.

We measured the shared genetic drift between each newly sequenced individual (X) and other dogs and wolves (Y) since their separation from an outgroup, Dhole, using *f*_*3*_(*X*, *Y*; *Dhole*) (Raghavan et al., 2014, Wang et al., 2019a) and found a similar pattern as above, where the Zhejiang wolf shares the most genetic drift with gray wolves from Guizhou (Figure S5). We then used D-statistics (Patterson et al., 2012) of the form *D*(*Fox*, *Test*; *X*, *Y*), where X and Y are previously published wolves, to formally test the relationship these historical wolves have with different wolf populations. For Guizhou and Zhejiang wolves, we find that they share more alleles with the SC gray wolves (Shanxi and China_X) than with all other wolves, as *D*(*Fox*, *W12_Guizhou/W13_Guizhou/W7_Zhejiang*; *X*, *SC*) > 0 (9.8 < Z < 38.0, Table S3). The Jiangxi wolf shares more allele with the Tibet and Qinghai gray wolves than with other wolves, as *D*(*Fox*, *W2_Jiangxi*; *X*, *Tibet/Qinghai*) > 0 (11.9 < Z < 27.6, Table S3), whereas Jilin and Heilongjiang wolves share the most alleles with NA gray wolves. These results support our above-mentioned analyses, again grouping the Guizhou and Zhejiang wolves with the SC wolves, the Jiangxi wolf with the Tibetan wolves, and the Jilin and Heilongjiang wolves with the NA wolves.

In summary, our results revealed that the lowland Chinese wolves (Fan et al., 2016) consisted of two major populations: SC and NA wolves. Gray wolves from Zhejiang and Guizhou group most closely with and share the most genetic drift with SC wolves, which includes present-day populations in Shanxi and China_X. The Jilin and Heilongjiang gray wolves share the most genetic similarity to the NA gray wolves, which is the other clade in Northern China and Eastern Russia.

### Testing for Admixture in Gray Wolves

Using *D*(*Fox*, *X*; *Test*, *Y*), we find that all gray wolves (X) share more alleles with the Guizhou, Jilin, and Heilongjiang wolves (Test) than with the gray wolves from Tibet and Qinghai, i.e., *D*(*Fox*, *X*; *Test*, *Qinghai/Tibet*) *< 0* (Table S5). The Jiangxi wolf shares more alleles with the Tibet and Qinghai wolves than with the other wolves (Table S3), and here, we find that the Tibet and Qinghai wolves share more alleles with the Jiangxi wolf, i.e., *D*(*Fox*, *Qinghai/Tibet*; *W2_Jiangxi*, *X*) *< 0* (−34.5 < Z < −15.4, Table S5), emphasizing that the Jiangxi, Tibet, and Qinghai wolves form a clade. However, whereas *D*(*Fox*, *X*; *W2_Jiangxi*, *Qinghai*)*∼0* (−2.3 < Z < 1.8, Table S5), indicating a symmetric relationship as expected for the Jiangxi and Qinghai wolves forming a clade, we observe *D*(*Fox*, *X*; *W12_Jiangxi*, *Tibet*) *< 0* (Table S5), suggesting that the Jiangxi wolf has a connection to other wolves relative to the Tibetan wolf. Thus, we observe that the Tibet and Qinghai gray wolves act as an outgroup to most SC and NA gray wolf populations, and although the Jiangxi wolf forms a clade with the Tibet and Qinghai gray wolves, the Jiangxi wolf also shows connections to non-Tibetan wolf populations. To test for admixture between the SC, NA, and Tibetan wolves, we used *f3*(*Test*; *X*, *Y*), where a significantly negative value (Z < −3) suggests that the Test population is a mixture of ancestry related to X and Y, two other wolf populations. Testing all combinations as both a source population and the admixed population, we found that *f3*(*W12_Jiangxi/Qinghai*; *Tibetan*, *SC/NA*) *< 0* (−19.101 < Z < −9.141, Table S4), suggesting that both the Jiangxi and Qinghai wolves show evidence of ancestry from populations related to both Tibetan and SC gray wolves, explaining why the Jiangxi gray wolf shares a connection to non-Tibetan gray wolf populations.

In contrast to all other gray wolves from China, the Zhejiang wolf shows a markedly different pattern, where all other wolf populations, including the Tibetan and Qinghai gray wolves and more distantly related wolves from further west, form a clade with each other relative to the Zhejiang wolf. That is, we observe that *D*(*Fox*, *X*; *W7_Zhejiang*, *Y*) *> 0* (8.0 < Z < 81.3, Table S5), where X and Y are all other gray wolves, including the Taimyr. Earlier, we found that *D*(*Fox*, *W7_Zhejiang*; *X*, *Shanxi/China_X*) *> 0* (Table S3), indicating that the Zhejiang wolf shows connections to the wolves from Shanxi and China_X. These results suggest that the Zhejiang wolf shows a close relationship to gray wolves from Shanxi and China_X, but that this wolf also possesses an ancestral component that is older than the common ancestral population of the Taimyr and all other gray wolves. The error rate for the Zhejiang wolf (0.4%) is higher than that estimated for other wolves sequenced in this study (0.1%–0.2%), likely because of its low coverage (Table 1). After simulating an error rate similar to that observed for the Zhejiang wolf in these other wolves, we find that our results remain consistent with our previous results. That is, the Zhejiang wolf shows a distinct pattern from that observed in other wolves for both lower and higher error rates.

We use the genomic data from canids typically outgroup to all wolves and dogs, the Dhole, Jackal, Coyote, and Red wolf, to understand how deeply the old component found in the Zhejiang wolf separated from other canid populations. Other canids separated from wolf populations very early, with the Dhole diverging earliest, followed by the Jackal and most recently the Coyote (Lindblad-Toh et al., 2005, Macdonald and Sillero-Zubiri, 2004, Wayne et al., 1997). The Red wolf is genetically very similar to the Coyote and shows substantial gene flow from gray wolves (vonHoldt et al., 2016, vonHoldt et al., 2011). First, comparing the Jackal to wolves (X) and the Coyote, we find that for all wolves but the Zhejiang wolf, *D*(*Fox*, *Jackal*; *X*, *Coyote*) ranges from −0.042 to −0.017 (−16.5 < Z < −6.4, Table S6), indicating a connection between the Jackal and gray wolves. We find the reverse for the Zhejiang wolf, however, where the Jackal shares more alleles with the Coyote than with the Zhejiang wolf, i.e., *D*(*Fox*, *Jackal*; *W7_Zhejiang*, *Coyote*)*=0*.*134* (Z = 34.1, Table S6). We find similar results replacing the Coyote with the Red wolf. The large contrast between the results for the Zhejiang wolf compared with other gray wolves suggests that the old component came from a population that diverged deeply in the past, who separated before the common ancestor of jackals and coyotes.

We also observe that for all gray wolves, we find *D*(*Fox*, *Dhole*; *X*, *Jackal*) > 0 (Table S6), suggesting that gray wolves share a deep lineage older than the separation of the Jackal and Dhole or that there is a direct genetic connection between the Jackal and Dhole. However, whereas D ranges from 0.004 to 0.028 (4.5 < Z < 7.9, Table S6) for most gray wolves, using the Zhejiang wolf greatly increases the D value to 0.117 (Z = 20.2, Table S6). We find that *D*(*Fox*, *Dhole*; *W7_Zhejiang*, *Jackal*) remains significantly positive (Z = 12.2) using only transversions, suggesting that the result for the Zhejiang wolf is not related to aDNA damage and likely reflects an unusual admixture history. If the Zhejiang wolf was no different from other gray wolves, especially the two Guizhou individuals to which they share the closest relationship (Table S5), we would expect to find that *D*(*Fox*, *Dhole*; *X*, *Zhejiang*)*∼0*, which would indicate that the Zhejiang wolf and other gray wolves are similarly related to the Dhole. However, we observe that *D*(*Fox*, *Dhole*; *X*, *W7_Zhejiang*) < 0 (−22.4 < Z < −11.7, Table S6), indicating that the Dhole shares more alleles with other gray wolves than with the Zhejiang wolf. These patterns suggest that the ancestral component found in the Zhejiang wolf came from a population that diverged earlier than the common ancestor of the Jackal and Dhole, which is older than the separation of the Jackal and Dhole from wolves, suggesting that the Zhejiang wolf possesses very deep ancestry not found in other gray wolves.

**Figure 2:**
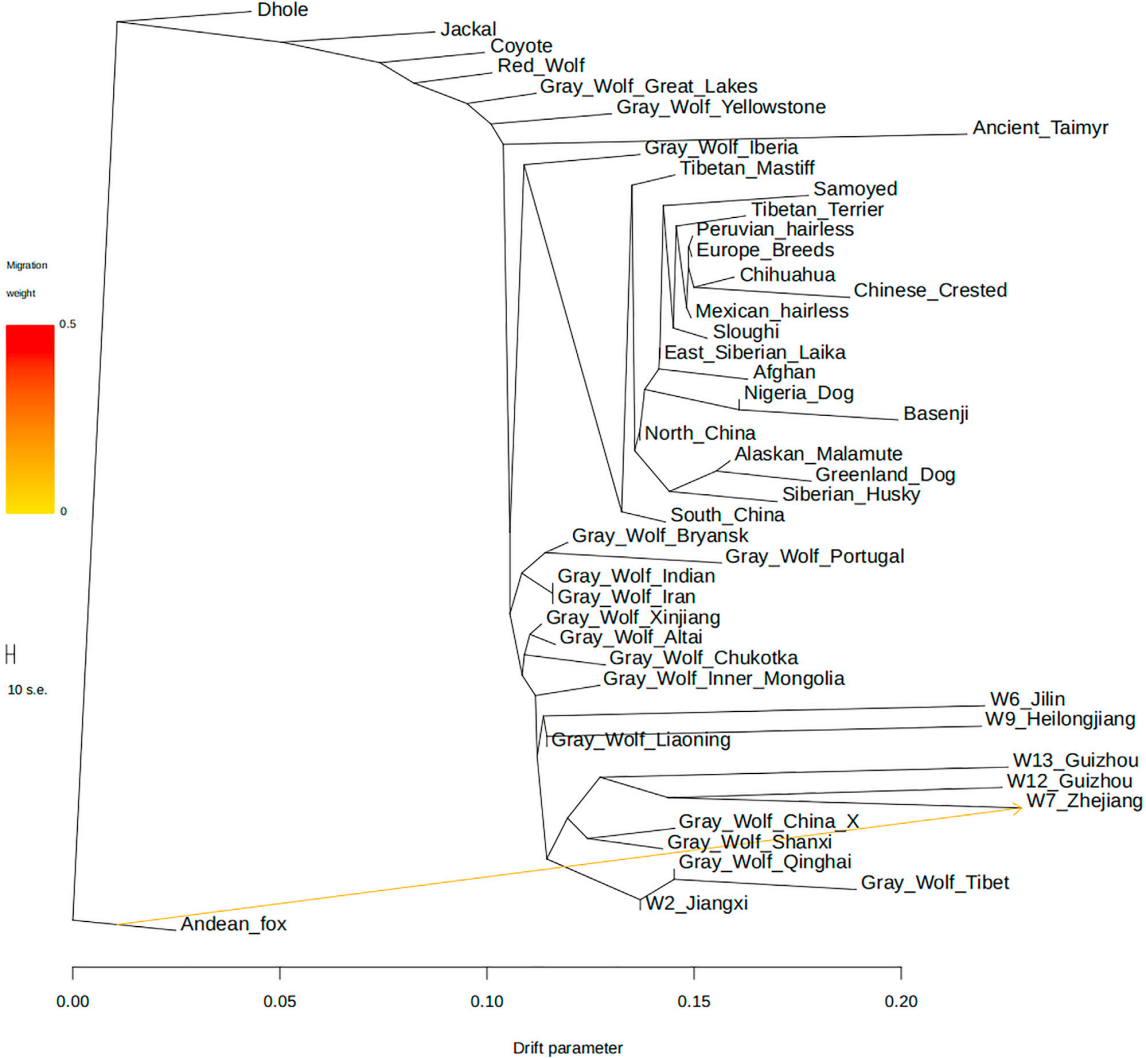
The Maximum Likelihood Tree Based on TreeMix with m = 1 The scale bar shows 10 times the average standard error of the entries in the sample covariance matrix. We have used the prefix “Gray_Wolf_” for highlighting gray wolves.

Using the tree model with no admixture from TreeMix (Figure S6), we visualized the matrix of residuals (Figure S7) to determine how the estimated genetic relationship between each pair of canids fit the model. A high residual indicates that the pair does not fit the tree model and may be candidates for an admixture event. We find two candidate admixture events, the first between the Andean fox and Zhejiang wolf (Figure 2) and the second between gray wolves from Portugal and Iberia. In an ML tree allowing two admixture events, admixture from the lineage leading to the Andean fox to the lineage leading to the Zhejiang wolf is included, whereas gray wolves from Portugal and Iberia are grouped into the same cluster (Figure S8). Admixture between the outgroup Andean fox and the Zhejiang wolf supports our conclusions from the D-statistic analysis (Tables S5 and S6), in which the Zhejiang wolf possesses an ancestral component that came from a population that diverged earlier than the Jackal or Dhole did from wolves. The estimated value of the migration event in the Zhejiang individual is 12.3% ± 0.4% (p = 2.2 × 10^−308^). In the TreeMix analysis, we used the Andean fox as an outgroup, whose distance from the included canids would result in weak phylogenetic constraints. In addition, we also used the F4-ratio test to estimate the proportion of this deep ancestry, and as it is older than the separation of the Jackal and Dhole, we used an unrooted phylogeny where the Fox is used as a proxy for the source of the deep ancestry (Figure S9). Thus, we estimate the proportion of ancestry related to the Fox, which is given by:

$$\frac{f_{4}\left(Dhole,\;Jackal,\;Coyote,\;X\right)}{f_{4}\left(Dhole,\;Jackal,\;Coyote,\;Andean\;Fox\right)}\text{,}$$

where X is each gray wolf in turn.

Using this method, we found that the estimated admixture proportion of the deep ancestry for the Zhejiang wolf is 11.7% ± 0.5%, whereas all other gray wolves have an estimated admixture proportion close to zero. Similarly, in the qpGraph analysis, we estimated an admixture proportion for the deep ancestry in W7_Zhejiang of 14% (Figures S10–S14). Thus the TreeMix, F4-ratio and admixture graph analyses all support the presence of gene flow from an ancient canid population into the ancestors of the Zhejiang wolf.

### Conclusion

The distribution of gray wolves in East Asia is controversial because some studies have claimed that gray wolves never existed (Callaway, 2013, Larson and Fuller, 2014) or are now extinct in SC (Lau et al., 2010), whereas others sources, especially those based on Chinese literature, have stated that they are present across all of mainland China (Wang et al., 2016b). In this study, we provide the first comparative genomic analysis of gray wolves from East Asia, focusing particularly on wolves from SC, where some believed no gray wolves were distributed (Callan et al., 2013, Larson and Fuller, 2014). Previously, Asian wolves could be divided into two populations: Tibetan gray wolves (*Canis lupus chanco*) and Chinese lowland wolves (Fan et al., 2016, Zhang et al., 2014). Here, using ancient genome-wide data, we reconstruct the phylogeny and evaluate the population structure and shared genetic drift between East Asian gray wolves to show that they form three major groups, which we call Southern China (SC), Northern Asia (NA), and Tibetan, based on their geographic distribution. Interestingly, specimens from SC gray wolves were all collected from 1963 to 1988. Our results highlight that the population in SC is endemic, and with the fast growing economic development of China, it is paramount to protect and restore their ecological habitat. Through our study, we also emphasize the value of using paleogenomic approaches to study the numerous museum specimens available (Min-Shan Ko et al., 2018, Roy et al., 1994), and we address the importance of using population genomics to determine current or future conservation efforts.

Finally, our analyses show that admixture played a large role between the different Asian wolves, and we highlight two instances here. First, we find that a wolf as far southeast as Jiangxi province shows evidence of being a mixture of Tibetan-related wolves and other wolves in China. Second, we traced an unusual admixture event in the Zhejiang wolf. In many analyses, this wolf behaved similarly to other gray wolves in China, particularly those from SC. However, tests of admixture suggest that the Zhejiang wolf shows gene flow from a canid that diverged earlier than the separation of wolves and jackals. D-statistic analysis suggests that this may be from the Dhole, a species distributed in SC and Southeast Asia (Iyengar et al., 2005), or another canid that separated earlier than the divergence between wolves and the Dhole. Whatever the source of this ancestry, estimates of the admixture proportion from this deeply diverging population are estimated to be ∼12%–14%. Our results, taken together with previous research (Gopalakrishnan et al., 2018), reveal that canids are an ideal system in which to study how gene flow can shape speciation in a genus, and highlight the need for greater study of ancient gray wolf populations.

### Limitations of Study

Although our results showed support for a SC gray wolf population and strong evidence for interspecific gene flow to the W7_Zhejiang wolf, the source of the interspecific gene flow into W7_Zhejiang is unclear. The availability of few specimens makes sampling in appropriate regions difficult, and where specimens are available, obtaining high coverage genomic data is not easy owing to natural degradation processes and loss of genetic material from museum storage methods. More samples are needed in future studies to further understand the admixture dynamics in canids.

## Methods

All methods can be found in the accompanying Transparent Methods supplemental file.

## Data and Code Availability

The accession number for the sequence data of six gray wolf genomes in this paper is GSA: PRJCA001135 and the SRA: PRJEB34110. The SNP set is available on the iDog database (http://bigd.big.ac.cn/idog/).

## Supplemental Information

Document S1. Transparent Methods, Figures S1–S14, and Tables S1–S6

## References

[bib1] Auton A., Li Y.R., Kidd J., Oliveira K., Nadel J., Holloway J.K., Hayward J.J., Cohen P.E., Greally J.M., Wang J. (2013). Genetic recombination is targeted towards gene promoter regions in dogs. PLoS Genet..

[bib2] Botigué L.R., Song S., Scheu A., Gopalan S., Pendleton A.L., Oetjens M., Taravella A.M., Seregély T., Zeeb-Lanz A., Arbogast R.-M. (2017). Ancient European dog genomes reveal continuity since the Early Neolithic. Nat. Commun..

[bib3] Briggs A.W., Stenzel U., Johnson P.L., Green R.E., Kelso J., Prufer K., Meyer M., Krause J., Ronan M.T., Lachmann M. (2007). Patterns of damage in genomic DNA sequences from a Neandertal. Proc. Natl. Acad. Sci. U S A.

[bib4] Callan R., Nibbelink N.P., Rooney T.P., Wiedenhoeft J.E., Wydeven A.P. (2013). Recolonizing wolves trigger a trophic cascade in Wisconsin (USA). J. Ecol..

[bib5] Callaway E. (2013). Dog genetics spur scientific spat. Nature.

[bib6] Carmichael L.E., Nagy J.A., Larter N.C., Strobeck C. (2001). Prey specialization may influence patterns of gene flow in wolves of the Canadian Northwest. Mol. Ecol..

[bib7] Dabney J., Knapp M., Glocke I., Gansauge M.T., Weihmann A., Nickel B., Valdiosera C., Garcia N., Paabo S., Arsuaga J.L. (2013). Complete mitochondrial genome sequence of a Middle Pleistocene cave bear reconstructed from ultrashort DNA fragments. Proc. Natl. Acad. Sci. U S A.

[bib8] Decker B., Davis B.W., Rimbault M., Long A.H., Karlins E., Jagannathan V., Reiman R., Parker H.G., Drogemuller C., Corneveaux J.J. (2015). Comparison against 186 canid whole-genome sequences reveals survival strategies of an ancient clonally transmissible canine tumor. Genome Res..

[bib9] DePristo M.A., Banks E., Poplin R., Garimella K.V., Maguire J.R., Hartl C., Philippakis A.A., del Angel G., Rivas M.A., Hanna M. (2011). A framework for variation discovery and genotyping using next-generation DNA sequencing data. Nat. Genet..

[bib10] Fan Z., Silva P., Gronau I., Wang S., Armero A.S., Schweizer R.M., Ramirez O., Pollinger J., Galaverni M., Ortega Del-Vecchyo D. (2016). Worldwide patterns of genomic variation and admixture in gray wolves. Genome Res..

[bib11] Frantz L.A., Mullin V.E., Pionnier-Capitan M., Lebrasseur O., Ollivier M., Perri A., Linderholm A., Mattiangeli V., Teasdale M.D., Dimopoulos E.A. (2016). Genomic and archaeological evidence suggest a dual origin of domestic dogs. Science.

[bib12] Freedman A.H., Gronau I., Schweizer R.M., Ortega-Del Vecchyo D., Han E., Silva P.M., Galaverni M., Fan Z., Marx P., Lorente-Galdos B. (2014). Genome sequencing highlights the dynamic early history of dogs. PLoS Genet..

[bib13] Geffen E., Anderson M.J., Wayne R.K. (2004). Climate and habitat barriers to dispersal in the highly mobile grey wolf. Mol. Ecol..

[bib14] Gopalakrishnan S., Sinding M.S., Ramos-Madrigal J., Niemann J., Samaniego Castruita J.A., Vieira F.G., Caroe C., Montero M.M., Kuderna L., Serres A. (2018). Interspecific gene flow shaped the evolution of the genus canis. Curr. Biol..

[bib15] Iyengar A., Babu V.N., Hedges S., Venkataraman A.B., Maclean N., Morin P.A. (2005). Phylogeography, genetic structure, and diversity in the dhole (*Cuon alpinus*). Mol. Ecol..

[bib16] Kircher M., Sawyer S., Meyer M. (2012). Double indexing overcomes inaccuracies in multiplex sequencing on the Illumina platform. Nucleic Acids Res..

[bib17] Larson G., Fuller D.Q. (2014). The evolution of animal domestication. Annu. Rev. Ecol. Evol. Syst..

[bib18] Lau M.W.N., Fellowes J.R., Chan B.P.L. (2010). Carnivores (Mammalia: Carnivora) in South China: a status review with notes on the commercial trade. Mamm. Rev..

[bib19] Lindblad-Toh K., Wade C.M., Mikkelsen T.S., Karlsson E.K., Jaffe D.B., Kamal M., Clamp M., Chang J.L., Kulbokas E.J., Zody M.C. (2005). Genome sequence, comparative analysis and haplotype structure of the domestic dog. Nature.

[bib20] Liu Y.H., Wang L., Xu T., Guo X., Li Y., Yin T.T., Yang H.C., Hu Y., Adeola A.C., Sanke O.J. (2018). Whole-genome sequencing of african dogs provides insights into adaptations against tropical parasites. Mol. Biol. Evol..

[bib21] Loog L., Thalmann O., Sinding M.-H.S., Schuenemann V.J., Perri A., Germonpre M., Bocherens H., Witt K.E., Samaniego Castruita J.A., Velasco M.S. (2018). Modern wolves trace their origin to a late Pleistocene expansion from Beringia. bioRxiv.

[bib22] Macdonald D.W., Sillero-Zubiri C. (2004). Biology and Conservation of Wild Canids.

[bib23] Marsden C.D., Ortega-Del Vecchyo D., O'Brien D.P., Taylor J.F., Ramirez O., Vila C., Marques-Bonet T., Schnabel R.D., Wayne R.K., Lohmueller K.E. (2016). Bottlenecks and selective sweeps during domestication have increased deleterious genetic variation in dogs. Proc. Natl. Acad. Sci. U S A.

[bib24] Meyer M., Kircher M. (2010). Illumina sequencing library preparation for highly multiplexed target capture and sequencing. Cold Spring Harb Protoc..

[bib25] Min-Shan Ko A., Zhang Y., Yang M.A., Hu Y., Cao P., Feng X., Zhang L., Wei F., Fu Q. (2018). Mitochondrial genome of a 22,000-year-old giant panda from southern China reveals a new panda lineage. Curr. Biol..

[bib26] Monzon J., Kays R., Dykhuizen D.E. (2014). Assessment of coyote-wolf-dog admixture using ancestry-informative diagnostic SNPs. Mol. Ecol..

[bib27] Patterson N., Moorjani P., Luo Y., Mallick S., Rohland N., Zhan Y., Genschoreck T., Webster T., Reich D. (2012). Ancient admixture in human history. Genetics.

[bib28] Patterson N., Price A.L., Reich D. (2006). Population structure and eigenanalysis. PLoS Genet..

[bib29] Pickrell J.K., Pritchard J.K. (2012). Inference of population splits and mixtures from genome-wide allele frequency data. PLoS Genet..

[bib30] Pilot M., Greco C., vonHoldt B.M., Jedrzejewska B., Randi E., Jedrzejewski W., Sidorovich V.E., Ostrander E.A., Wayne R.K. (2014). Genome-wide signatures of population bottlenecks and diversifying selection in European wolves. Heredity (Edinb.).

[bib31] Pilot M., Jedrzejewski W., Branicki W., Sidorovich V.E., Jedrzejewska B., Stachura K., Funk S.M. (2006). Ecological factors influence population genetic structure of European grey wolves. Mol. Ecol..

[bib32] Pocock R.I., Sewel R.B.S. (1941). The Fauna of British India Including Ceylon and Burma. Mammalia, Vol. II. Carnivora.

[bib33] Raghavan M., Skoglund P., Graf K.E., Metspalu M., Albrechtsen A., Moltke I., Rasmussen S., Stafford T.W., Orlando L., Metspalu E. (2014). Upper Palaeolithic Siberian genome reveals dual ancestry of Native Americans. Nature.

[bib34] Roy M.S., Girman D.J., Taylor A.C., Wayne R.K. (1994). The use of museum specimens to reconstruct the genetic-variability and relationships of extinct populations. Experientia.

[bib35] Shannon L.M., Boyko R.H., Castelhano M., Corey E., Hayward J.J., McLean C., White M.E., Abi Said M., Anita B.A., Bondjengo N.I. (2015). Genetic structure in village dogs reveals a Central Asian domestication origin. Proc. Natl. Acad. Sci. U S A.

[bib36] Skoglund P., Ersmark E., Palkopoulou E., Dalen L. (2015). Ancient wolf genome reveals an early divergence of domestic dog ancestors and admixture into high-latitude breeds. Curr. Biol..

[bib37] Smith A.T., Xie Y. (2014). A Guide to the Mammals of China.

[bib38] Tang B., Zhou Q., Dong L., Li W., Zhang X., Lan L., Zhai S., Xiao J., Zhang Z., Bao Y. (2019). iDog: an integrated resource for domestic dogs and wild canids. Nucleic Acids Res..

[bib39] Thalmann O., Shapiro B., Cui P., Schuenemann V.J., Sawyer S.K., Greenfield D.L., Germonpre M.B., Sablin M.V., Lopez-Giraldez F., Domingo-Roura X. (2013). Complete mitochondrial genomes of ancient canids suggest a European origin of domestic dogs. Science.

[bib40] vonHoldt B.M., Cahill J.A., Fan Z., Gronau I., Robinson J., Pollinger J.P., Shapiro B., Wall J., Wayne R.K. (2016). Whole-genome sequence analysis shows that two endemic species of North American wolf are admixtures of the coyote and gray wolf. Sci. Adv..

[bib41] vonHoldt B.M., Pollinger J.P., Earl D.A., Knowles J.C., Boyko A.R., Parker H., Geffen E., Pilot M., Jedrzejewski W., Jedrzejewska B. (2011). A genome-wide perspective on the evolutionary history of enigmatic wolf-like canids. Genome Res..

[bib42] Vonholdt B.M., Pollinger J.P., Lohmueller K.E., Han E., Parker H.G., Quignon P., Degenhardt J.D., Boyko A.R., Earl D.A., Auton A. (2010). Genome-wide SNP and haplotype analyses reveal a rich history underlying dog domestication. Nature.

[bib43] Wang G.D., Shao X.J., Bai B., Wang J.L., Wang X.B., Cao X., Liu Y.H., Wang X., Yin T.T., Zhang S.J. (2019). Structural variation during dog domestication: insights from gray wolf and dhole genomes. Natl. Sci. Rev..

[bib44] Wang G.D., Zhai W., Yang H.C., Wang L., Zhong L., Liu Y.H., Fan R.X., Yin T.T., Zhu C.L., Poyarkov A.D. (2016). Out of southern East Asia: the natural history of domestic dogs across the world. Cell Res..

[bib45] Wang G.D., Zhai W.W., Yang H.C., Fan R.X., Cao X., Zhong L., Wang L., Liu F., Wu H., Cheng L.G. (2013). The genomics of selection in dogs and the parallel evolution between dogs and humans. Nat. Commun..

[bib46] Wang L., Ma Y.P., Zhou Q.J., Zhang Y.P., Savolaimen P., Wang G.D. (2016). The geographical distribution of grey wolves (*Canis lupus*) in China: a systematic review. Zool. Res..

[bib47] Wang X., Zhou B.W., Yang M.A., Yin T.T., Chen F.L., Ommeh S.C., Esmailizadeh A., Turner M.M., Poyarkov A.D., Savolainen P. (2019). Canine transmissible venereal tumor genome reveals ancient introgression from coyotes to pre-contact dogs in North America. Cell Res..

[bib48] Wayne R.K., Geffen E., Girman D.J., Koepfli K.P., Lau L.M., Marshall C.R. (1997). Molecular systematics of the Canidae. Syst. Biol..

[bib49] Wayne R.K., Lehman N., Allard M.W., Honeycutt R.L. (1992). Mitochondrial-DNA variability of the gray wolf - genetic consequences of population decline and habitat fragmentation. Conserv. Biol..

[bib50] Wilson D.E., Reeder D.M. (2005). Mammal Species of the World: A Taxonomic and Geographic Reference.

[bib51] Young S.P., Goldman E.A., American Wildlife, F. (1944). The Wolves of North America. Part-II. Classification of Wolves.

[bib52] Zhang W., Fan Z., Han E., Hou R., Zhang L., Galaverni M., Huang J., Liu H., Silva P., Li P. (2014). Hypoxia adaptations in the grey wolf (*Canis lupus chanco*) from Qinghai-Tibet Plateau. PLoS Genet..

