# Supplementary material for: Genomic Approaches Reveal an Endemic Subpopulation of Gray Wolves in Southern China
Source: iScience. 2019 Sep 10;20:110–8. doi: 10.1016/j.isci.2019.09.008 (PMC6817678; doi:10.1016/j.isci.2019.09.008)
Supplement: Document S1. Transparent Methods, Figures S1–S14, and Tables S1–S6 [file mmc1.pdf]

ISCI, Volume *20*

Supplemental Information

Genomic Approaches Reveal an Endemic Subpopulation of Gray Wolves in Southern China

Guo-Dong Wang, Ming Zhang, Xuan Wang, Melinda A. Yang, Peng Cao, Feng Liu, Heng Lu, Xiaotian Feng, Pontus Skoglund, Lu Wang, Qiaomei Fu, and Ya-Ping Zhang

Supplemental Information

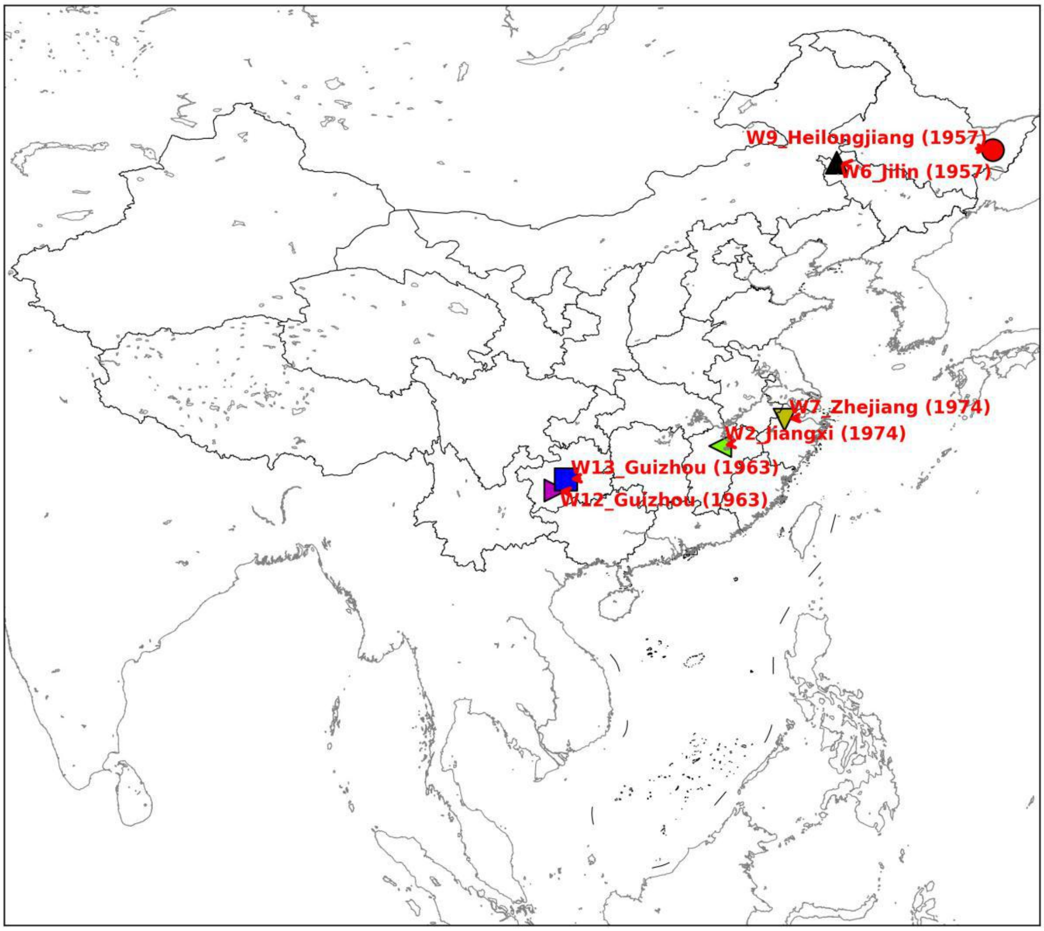

Figure S1. Geographical origin of six museum wolf skin specimens in China, two samples from Guizhou. The collection year is indicated in parantheses. Related to Figure 1.

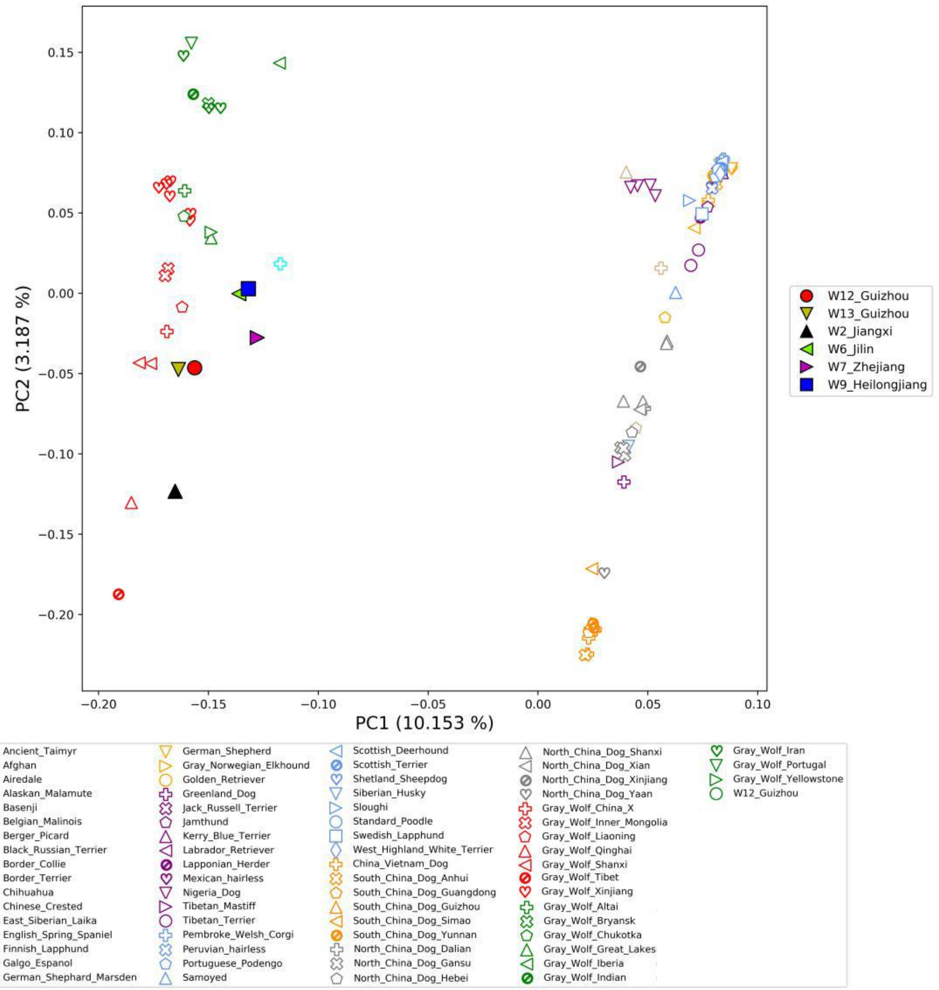

Figure S2. PCA analysis with wolf and dog populations. We have used the prefix "Gray_Wolf_" for highlighting specimens of gray wolves. Related to Figure 1.

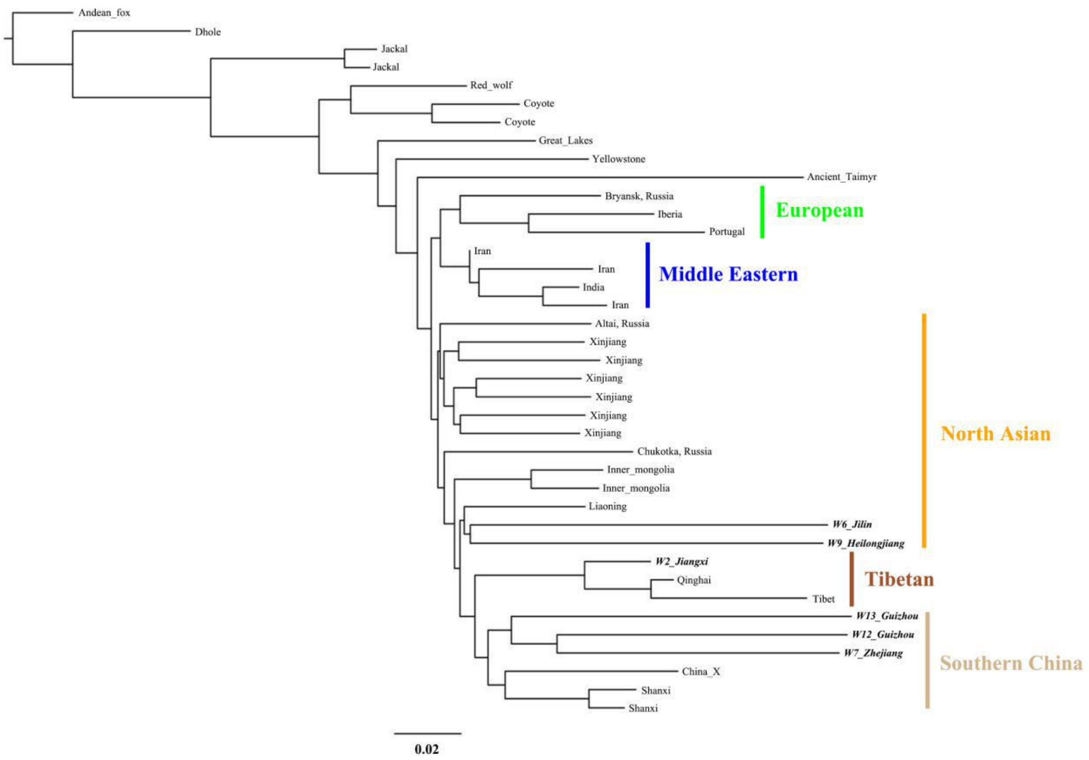

Figure S3. Neighbor-Joining tree including 39 canids without dogs. The Andean fox is the outgroup. The newly sequenced individuals are marked in bold and are italicized. Related to Figure 1.

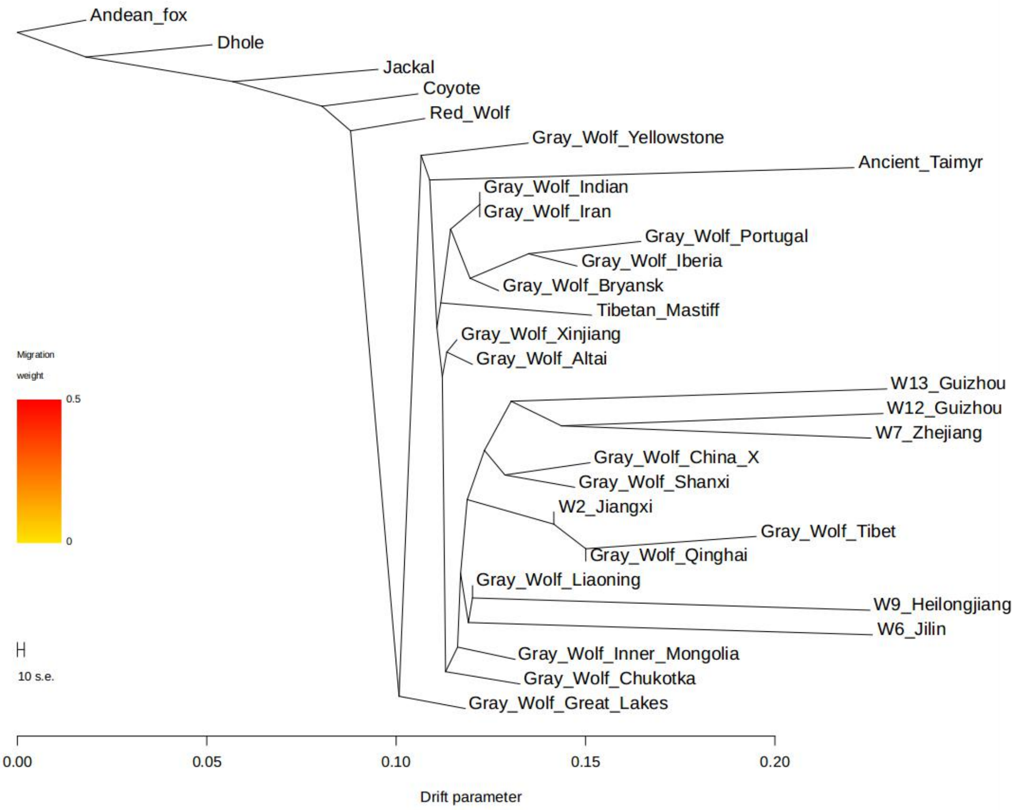

Figure S4. The maximum-likelihood tree based on TreeMix using 39 canids without dogs and m=0. The scale bar shows ten times the average standard error of the entries in the sample covariance matrix. Related to Figure 2.

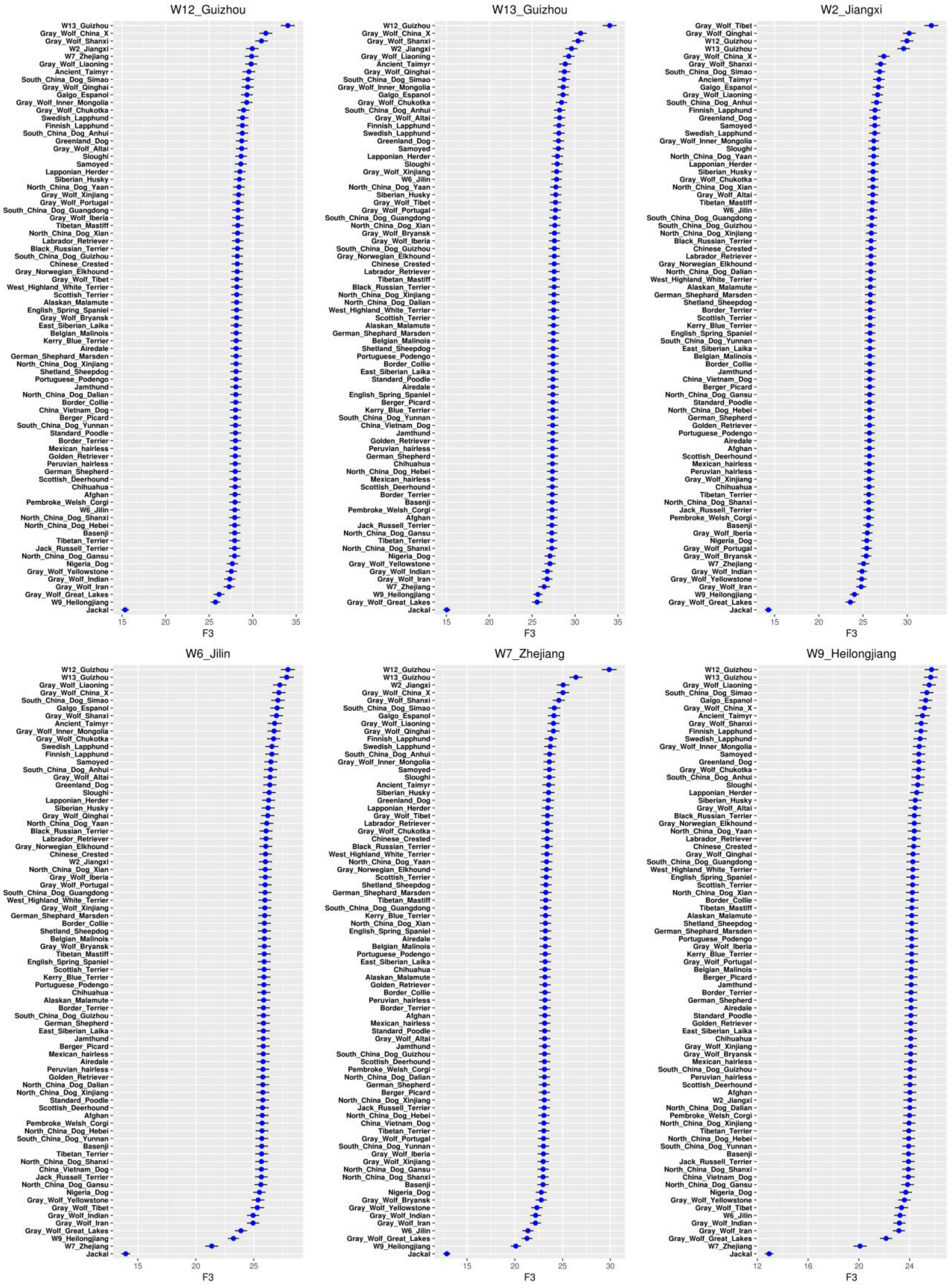

Figure S5. f3(Dhole; X, Y), where X is one of the six newly sequenced wolves and Y are other canids. We have used the prefix "Gray_Wolf_" for highlighting specimens of gray wolves. Related to Figure 1.

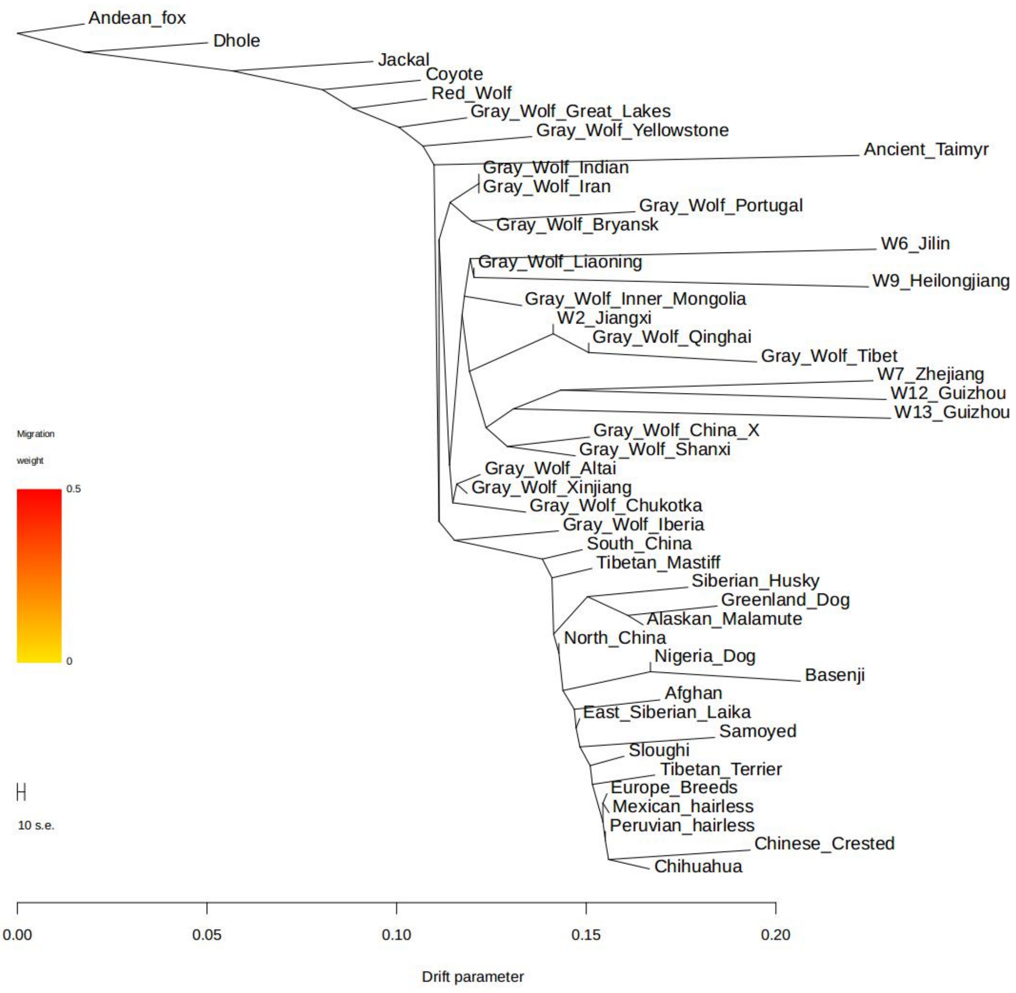

Figure S6. The maximum-likelihood tree based on TreeMix with m=0 for all canids. The scale bar shows ten times the average standard error of the entries in the sample covariance matrix. We have used the prefix "Gray_Wolf_" for highlighting specimens of gray wolves. Related to Figure 2.

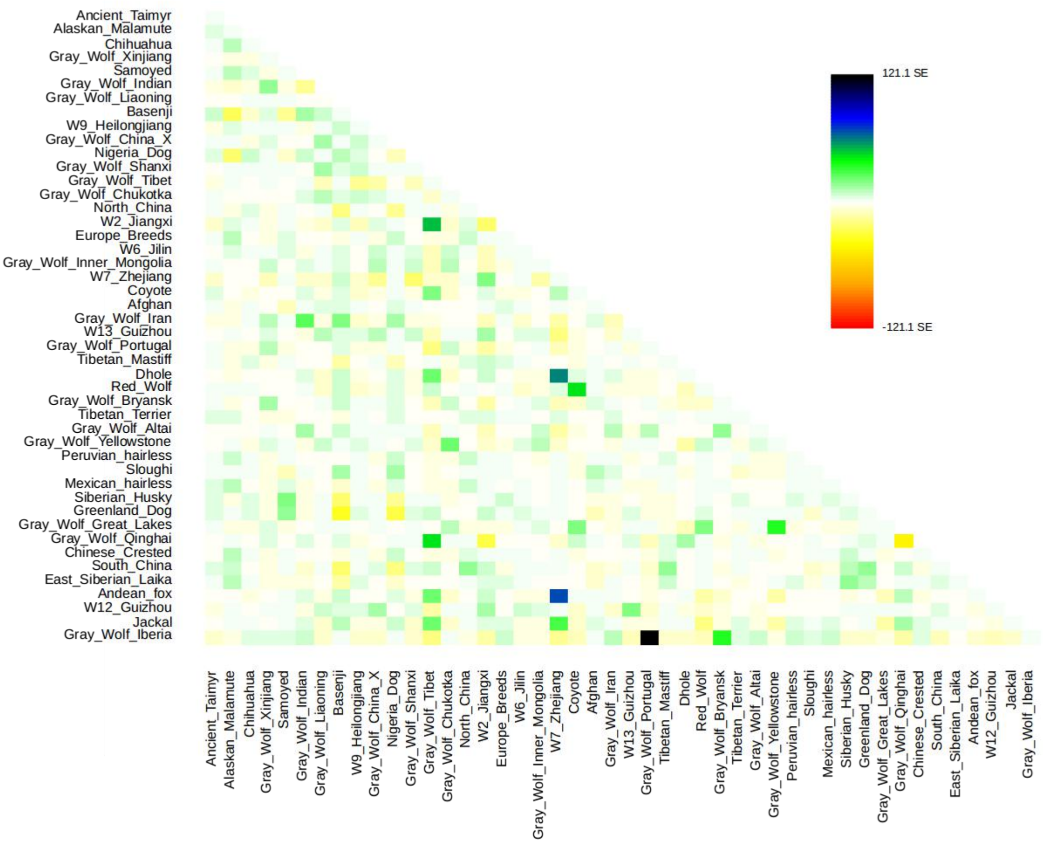

Figure S7. The residual fit from the maximum likelihood tree in Figure S6. This is determined by dividing the residual covariance between each pair of populations by the average standard error across all pairs. Colors are described in the palette on the right. Residuals above zero represent populations that are more closely related to each other in the data than in the best-fit tree, and thus are candidates for admixture events. We have used the prefix "Gray_Wolf_" for highlighting specimens of gray wolves. Related to Figure 2.

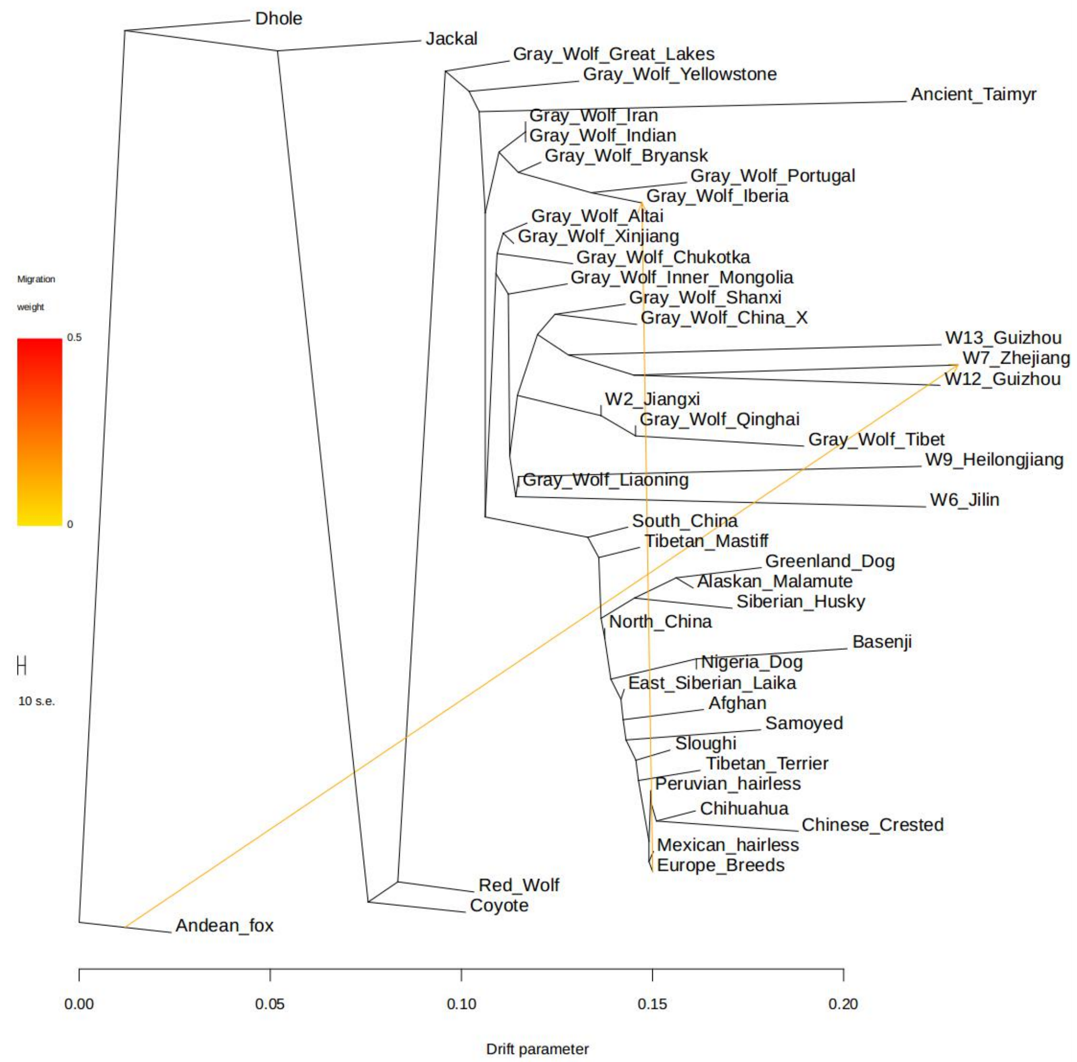

Figure S8. The maximum-likelihood tree based on TreeMix with m=2. The scale bar shows ten times the average standard error of the entries in the sample covariance matrix. We have used the prefix "Gray_Wolf_" for highlighting specimens of gray wolves. Related to Figure 2.

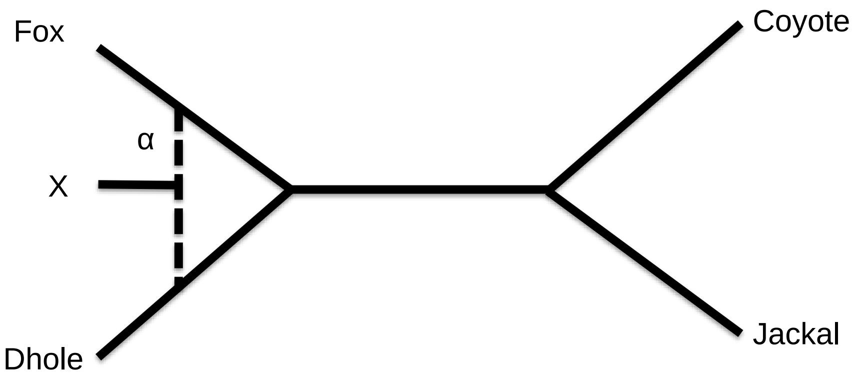

Figure S9. Unrooted tree used to estimate the archaic admixture proportion in the Zhejiang wolf. Related to Figure 2.

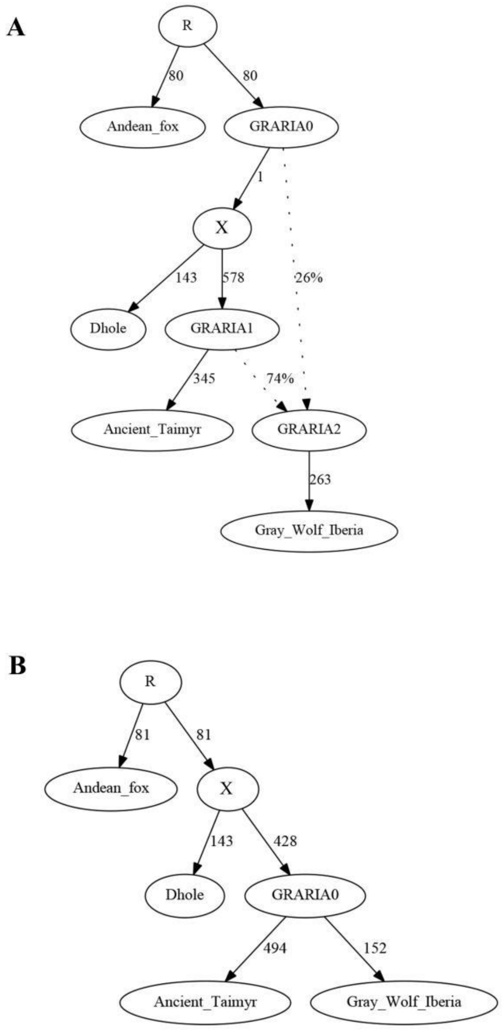

Figure S10. Basal Admixture Graph model with Gray_Wolf_Iberia added. Related to Figure 2.

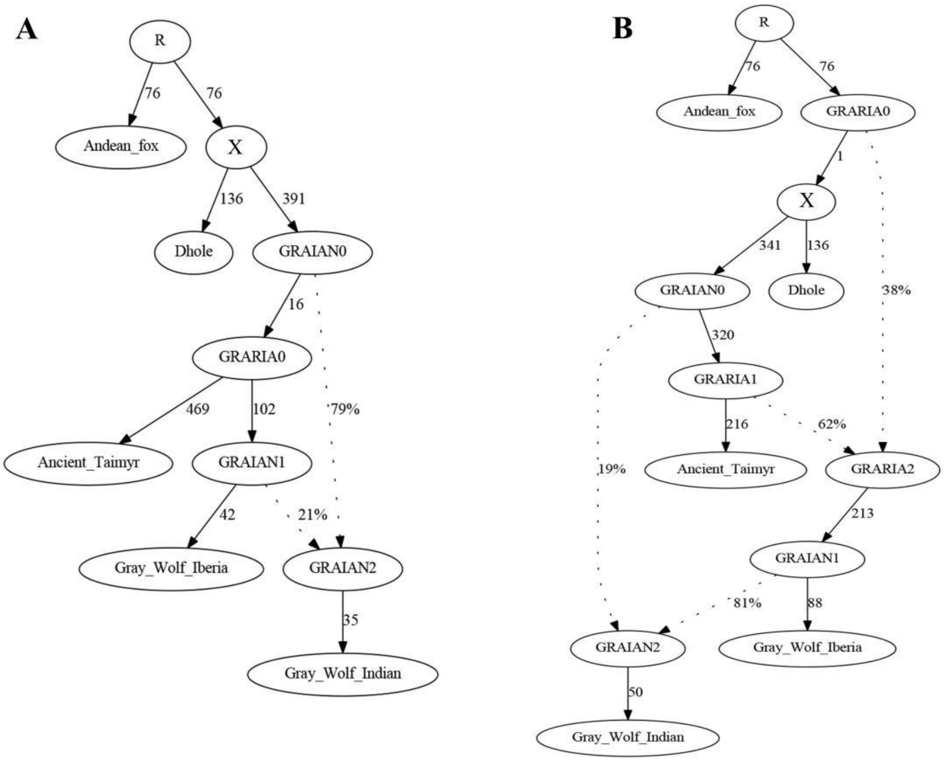

Figure S11. Adding Gray_Wolf_Indian to the graphs from Figure S10. Related to Figure 2.

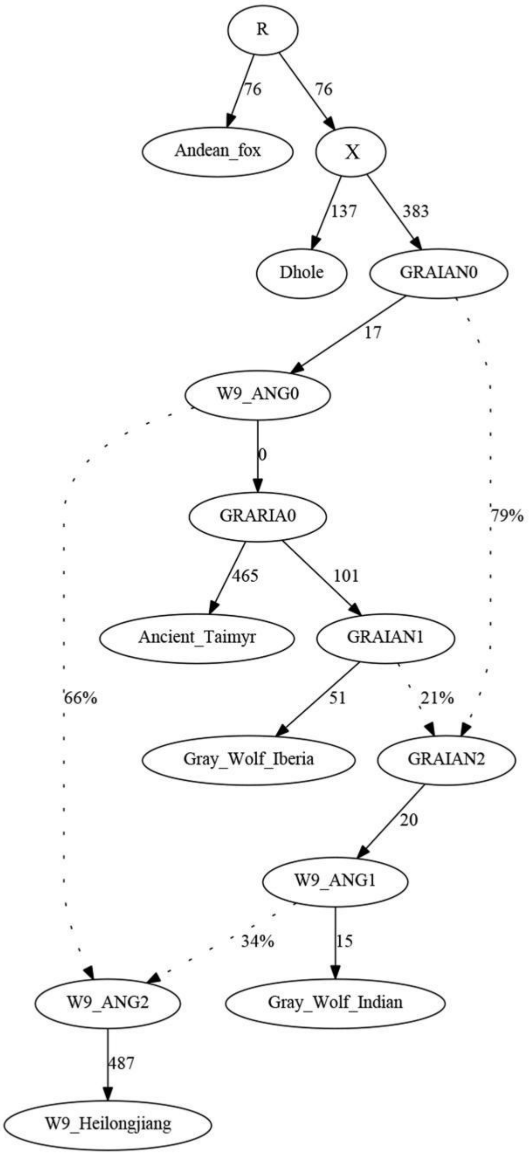

Figure S12. Adding W9_Heilongjiang to the graphs from Figure S11. Related to Figure 2.

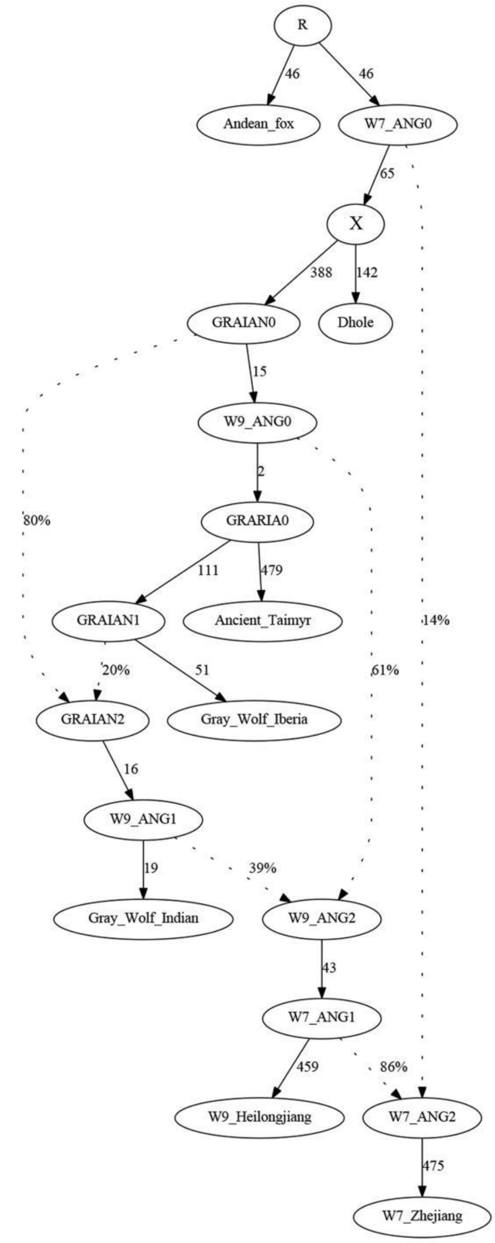

Figure S13. Adding W7_Zhejiang to the graphs from Figure S12. Related to Figure 2.

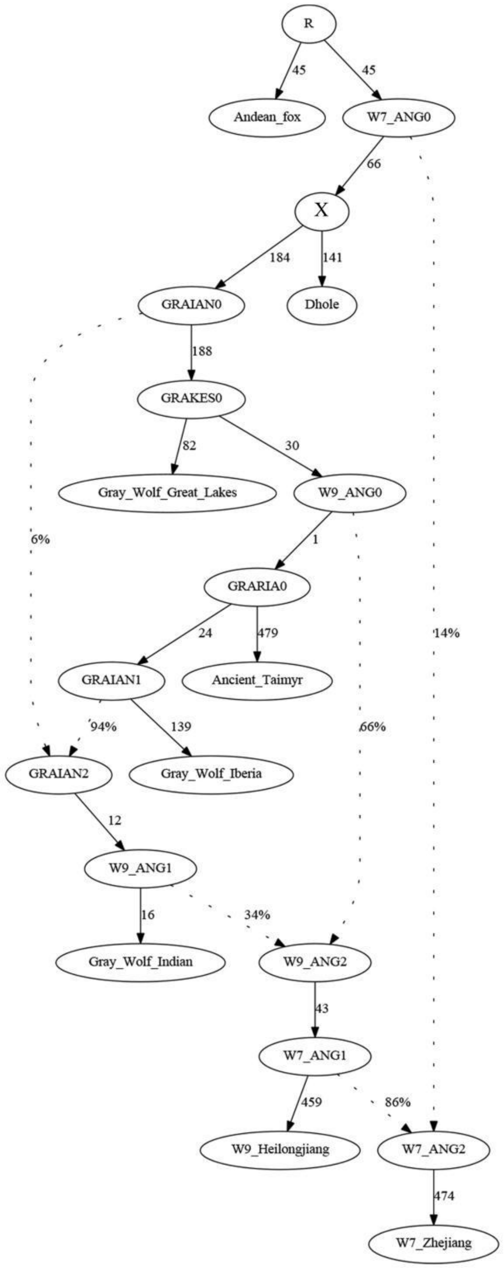

Figure S14. Adding Gray_Wolf_Great_Lakes to the graphs from Figure S13. Related to Figure 2.

Table S1. Sequencing metrics on the libraries of the samples. Related to Table 1.

Sample_ID	Lib_ID	Raw	Merged	&L30	Mapped	%Mapped	Unique	Average Length
W12_Guizhou	L4705	285343617	282720996	279349969	193639299	69	87020138	66
W12_Guizhou	L4706	122888988	121920909	120203042	81036195	67	37300930	66
W12_Guizhou	L4723	99422832	98301650	95950384	66689079	70	27296673	60
W12_Guizhou	L4724	129941007	128741838	125878134	86419489	69	35693699	60
W12_Guizhou	L4721	458823905	457178101	448236865	354599384	79	172415148	75
W12_Guizhou	L1236	220782482	214658250	212167619	172702204	81	83601122	82
W13_Guizhou	L4715	174435249	170656674	168612632	143289506	85	67178876	71
W13_Guizhou	L4733	100006948	97480812	96184443	79766884	83	37206113	74
W13_Guizhou	L4716	139660264	136843871	135476042	94368328	70	41458135	75
W13_Guizhou	L4734	72759420	71413179	70557705	48841322	69	20183735	72
W13_Guizhou	L1237	376669471	358779675	356919462	259792144	73	138338276	94
W2_Jiangxi	L4717	57032184	56165346	55929745	49470031	88	25470196	81
W2_Jiangxi	L4718	108657013	107230625	106675141	95573663	90	49006998	78
W2_Jiangxi	L4735	84038690	82911815	82368601	73430883	89	34211885	74
W2_Jiangxi	L4736	904760479	897952829	896171857	792391018	88	457142173	92
W2_Jiangxi	L1228	614619758	568519001	567900196	519132756	91	335201526	126
W6_Jilin	L4707	127681136	126097468	118289082	90384661	76	26902055	48
W6_Jilin	L4708	125430513	123589141	116027856	87251813	75	25879613	49
W6_Jilin	L4725	58051880	57192340	49981995	32368357	65	10635232	43
W6_Jilin	L4726	77378791	76219741	65107619	47902350	74	15267977	42
W6_Jilin	L1232	389136153	325919319	315703233	246562564	78	11324756	58
W7_Zhejiang	L4709	39112069	38593429	35402488	1843676	5	732974	57
W7_Zhejiang	L4710	35581720	35083335	32713276	1193424	4	395721	56
W7_Zhejiang	L4711	39015421	38438945	35639593	1833027	5	705431	64
W7_Zhejiang	L4712	39853260	39242507	36562343	2739467	7	1008169	63
W7_Zhejiang	L4727	39610530	39030578	32984973	2129260	6	796738	47
W7_Zhejiang	L4728	35207785	34640731	29296827	1317589	4	404382	46
W7_Zhejiang	L4729	44741369	44125280	37579861	2065001	5	683061	55
W7_Zhejiang	L4730	35964348	35467842	30198475	2576745	9	810242	54
W7_Zhejiang	L1233	216672870	196311964	188328108	8194354	4	324626	89
W9_Heilongjiang	L4713	60306027	59823823	56491103	48351903	86	17888738	51
W9_Heilongjiang	L4714	48275546	47733287	45095180	35539238	79	13910738	51
W9_Heilongjiang	L4731	78659917	78002396	69178887	58004190	84	20595472	44
W9_Heilongjiang	L4732	67266981	66511206	60235429	47951316	80	18366379	45
W9_Heilongjiang	L1234	146878870	129755983	125676741	53058660	42	1949475	71

Table S2. Genome information from public databases. Related to Table 1.

Species	Location	ID	Data sources
Fox	Andean	Andean_fox	Adam Auton et al., 2013, Plos Genetics
Dhole	Beijing Zoo	RUFZCHN00001	Guo-Dong Wang et al., 2018, National Science Review
Jackals	Krasnodar, Russia	AUR008537	Xuan Wang et al., 2019, Cell Research
	Krasnodar, Russia	AUR008538	Xuan Wang et al., 2019, Cell Research
Coyotes	Monterey area, Canada	LAT007000	Xuan Wang et al., 2019, Cell Research
	California, US	SAMN02921301	Bridgett M. vonHoldt et al., 2016, Science Advances
Red wolf	USA	SAMN02921317	Bridgett M. vonHoldt et al., 2016, Science Advances
Ancient wolf	Taimyr, Russia	Taimyr	Pontus Skoglund et al., 2015, Current Biology
Gray wolves	Great Lakes, USA	gwglw_RKW2455	Bridgett M. vonHoldt et al., 2016, Science Advances
	Yellowstone, USA	gwynp_RWK1547	Bridgett M. vonHoldt et al., 2016, Science Advances
	Iran	LUP004103	Xuan Wang et al., 2019, Cell Research
	Iran	LUP004107	Xuan Wang et al., 2019, Cell Research
	Iran	gwirw_RKW3073	Clare D. Marsden et al., 2016, PNAS
	India	SAMN02921311	Bridgett M. vonHoldt et al., 2016, Science Advances
	Bryansk, Russia	LUPWRUS00003	Guo-Dong Wang et al., 2013, Nature Communications
	Iberia	gwibe_XXWIB98	Laura R. Botigue et al., 2017, Nature Communications
	Portugal	gwprt_LOBO423	Clare D. Marsden et al., 2016, PNAS
	Shanxi, China	LUPZCHN00005	Guo-Dong Wang et al., 2016, Cell Research
	Shanxi, China	LUPZCHN00006	Guo-Dong Wang et al., 2016, Cell Research
	San Diego Zoo (from China)	gwcwz_RKW3916	Adam H. Freedman et al., 2014, Plos Genetics
	Qinghai, China	gwcwq_XinQH11	Wen-Ping Zhang, et al., 2014, Plos Genetics
	Tibet, China	gwcwt_XinTI09	Wen-Ping Zhang, et al., 2014, Plos Genetics
	Liaoning, China	LUPWCHN00003	Guo-Dong Wang et al., 2016, Cell Research
	Inner Mongolia, China	LUPWCHN00001	Guo-Dong Wang et al., 2013, Nature Communications
	Inner Mongolia, China	LUPZCHN00002	Guo-Dong Wang et al., 2016, Cell Research
	Chukotka, Russia	LUPWRUS00002	Guo-Dong Wang et al., 2013, Nature Communications
	Xinjiang, China	LUPWCHN00008	Guo-Dong Wang et al., 2016, Cell Research
	Xinjiang, China	LUPWCHN00009	Guo-Dong Wang et al., 2016, Cell Research
	Xinjiang, China	LUPWCHN00010	Guo-Dong Wang et al., 2016, Cell Research

	Xinjiang, China	LUPWCHN00013	Guo-Dong Wang et al., 2016, Cell Research
	Xinjiang, China	gwcwx_XinXJ24	Wen-Ping Zhang, et al., 2014, Plos Genetics
	Xinjiang, China	gwcwx_XinXJ30	Wen-Ping Zhang, et al., 2014, Plos Genetics
	Altai, Russia	LUPWRUS00001	Guo-Dong Wang et al., 2013, Nature Communications
	Afghan	FAMBAFG00001	Guo-Dong Wang et al., 2016, Cell Research
	Alaskan Malamute	FAMBALM00001	Guo-Dong Wang et al., 2016, Cell Research
	Belgian Malinois	FAMBBEM00001	Guo-Dong Wang et al., 2016, Cell Research
	Chihuahua	FAMBCHI00001	Guo-Dong Wang et al., 2016, Cell Research
	East Siberian Laika	FAMBESL00001	Guo-Dong Wang et al., 2016, Cell Research
	Finnish Lapphund	FAMBFIL00001	Guo-Dong Wang et al., 2016, Cell Research
	Galgo Español	FAMBGAL00001	Guo-Dong Wang et al., 2016, Cell Research
	Gray Norwegian Elkhound	FAMBGNE00001	Guo-Dong Wang et al., 2016, Cell Research
	Greenland dog	FAMBGRD00001	Guo-Dong Wang et al., 2016, Cell Research
	German Shepherd Dog	FAMBGSD00001	Guo-Dong Wang et al., 2016, Cell Research
	Jämthund	FAMBJAM00001	Guo-Dong Wang et al., 2016, Cell Research
	Lapponian Herder	FAMBLAH00001	Guo-Dong Wang et al., 2016, Cell Research
	Mexican hairless	FAMBMEN00001	Guo-Dong Wang et al., 2016, Cell Research
	Peruvian hairless	FAMBPEN00001	Guo-Dong Wang et al., 2016, Cell Research
	Samoyed	FAMBSAM00001	Guo-Dong Wang et al., 2016, Cell Research
	Siberian Husky	FAMBSIH00001	Guo-Dong Wang et al., 2016, Cell Research
	Sloughi	FAMBSLO00001	Guo-Dong Wang et al., 2016, Cell Research
	Swedish Lapphund	FAMBSWL00001	Guo-Dong Wang et al., 2016, Cell Research
Breed dogs	Tibetan Mastiff	FAMBTIM00001	Guo-Dong Wang et al., 2016, Cell Research
	Airedale	ddair_RS74411	D. Marsden Clare et al., 2016 PNAS
	Basenji	ddbas_RS80704	D. Marsden Clare et al., 2016 PNAS
	Border Collie	ddbdr_RS74410	D. Marsden Clare et al., 2016 PNAS
	Border Terrier	ddbdt_RS86407	D. Marsden Clare et al., 2016 PNAS
	Berger Picard	ddber_RS86405	D. Marsden Clare et al., 2016 PNAS
	Black Russian Terrier	ddbrt_RS86399	D. Marsden Clare et al., 2016 PNAS
	Chinese Crested	ddccr_RS88178	D. Marsden Clare et al., 2016 PNAS
	English Spring Spaniel	ddess_RS80702	D. Marsden Clare et al., 2016 PNAS
	Golden Retriever	ddgdr_RS86402	D. Marsden Clare et al., 2016 PNAS
	German Shephard Marsden	ddgsh_RS80703	D. Marsden Clare et al., 2016 PNAS
	Jack Russell Terrier	ddjrt_RS86400	D. Marsden Clare et al., 2016 PNAS
	Jack Russell Terrier	ddjrt_RS86404	D. Marsden Clare et al., 2016 PNAS
	Kerry Blue Terrier	ddkbt_RS74408	D. Marsden Clare et al., 2016 PNAS
	Labrador Retriever	ddlab_RS86398	D. Marsden Clare et al., 2016 PNAS
	Portuguese Podengo	ddppo_RS74409	D. Marsden Clare et al., 2016 PNAS
	Pembroke Welsh Corgi	ddpwc_RS73323	D. Marsden Clare et al., 2016 PNAS
	Pembroke Welsh Corgi	ddpwc_RS86409	D. Marsden Clare et al., 2016 PNAS

	Scottish Terrier	ddsct_RS86393	D. Marsden Clare et al., 2016 PNAS
	Scottish Deerhound	ddsdh_RS86401	D. Marsden Clare et al., 2016 PNAS
	Standard Poodle	ddspo_RS86408	D. Marsden Clare et al., 2016 PNAS
	Shetland Sheepdog	ddssd_RS88649	D. Marsden Clare et al., 2016 PNAS
	Tibetan Terrier	ddtbt_RS86403	D. Marsden Clare et al., 2016 PNAS
	Tibetan Terrier	ddtbt_RS86406	D. Marsden Clare et al., 2016 PNAS
	West Highland White Terrier	ddwhw_RS86397	D. Marsden Clare et al., 2016 PNAS
Village dogs	Xi'an, Shaanxi, China	FAMICHN00001	Guo-Dong Wang et al., 2013, Nature Communications
	Ya'an, Sichuan, China	FAMICHN00003	Guo-Dong Wang et al., 2013, Nature Communications
	Dalian, Liaoning, China	FAMICHN00004	Guo-Dong Wang et al., 2016, Cell Research
	Gansu, China	FAMICHN00005	Guo-Dong Wang et al., 2016, Cell Research
	Gansu, China	FAMICHN00006	Guo-Dong Wang et al., 2016, Cell Research
	Gansu, China	FAMICHN00007	Guo-Dong Wang et al., 2016, Cell Research
	Hebei, China	FAMICHN00012	Guo-Dong Wang et al., 2016, Cell Research
	Shanxi, China	FAMICHN00014	Guo-Dong Wang et al., 2016, Cell Research
	Shanxi, China	FAMICHN00015	Guo-Dong Wang et al., 2016, Cell Research
	Shaanxi, China	FAMICHN00016	Guo-Dong Wang et al., 2016, Cell Research
	Shaanxi, China	FAMICHN00017	Guo-Dong Wang et al., 2016, Cell Research
	Xinjiang, China	FAMICHN00019	Guo-Dong Wang et al., 2016, Cell Research
	Simao, Yunnan, China	FAMICHN00002	Guo-Dong Wang et al., 2013, Nature Communications
	Guangdong, China	FAMICHN00010	Guo-Dong Wang et al., 2016, Cell Research
	Guizhou, China	FAMICHN00011	Guo-Dong Wang et al., 2016, Cell Research
	Yunnan, China	FAMICHN00021	Guo-Dong Wang et al., 2016, Cell Research
	Yunnan, China	FAMICHN00023	Guo-Dong Wang et al., 2016, Cell Research
	Anhui, China	FAMICHN00025	Guo-Dong Wang et al., 2016, Cell Research
	Ibadan, Nigeria	FAMINGR00001	Guo-Dong Wang et al., 2016, Cell Research
	Ondo, Nigeria	FAMINGR00002	Guo-Dong Wang et al., 2016, Cell Research
	Uyo, Nigeria	FAMINGR00003	Guo-Dong Wang et al., 2016, Cell Research
	Taraba State, Nigeria	FAMINGR00004	Guo-Dong Wang et al., 2016, Cell Research
	China/Vietnam border	FAMIVNM00001	Guo-Dong Wang et al., 2016, Cell Research
	China/Vietnam border	FAMIVNM00002	Guo-Dong Wang et al., 2016, Cell Research
	China/Vietnam border	FAMIVNM00003	Guo-Dong Wang et al., 2016, Cell Research
	China/Vietnam border	FAMIVNM00004	Guo-Dong Wang et al., 2016, Cell Research
	China/Vietnam border	FAMIVNM00005	Guo-Dong Wang et al., 2016, Cell Research

Table S3. Z-scores for *D(Fox, Test; X, Y)*. Related to Figure 1 and Figure 2.

D(Andean_fox, W12_Guizhou; P3, P4)

X/Y	Ancient_Taimyr	Iran	Indian	Portugal	Inner_Mongolia	Liaoning	Xinjiang	Altai	Chukotka	Bryansk	Shanxi	China_X	Iberia	Qinghai	Tibet
Ancient_Taimyr		-0.7	0.0	5.8	25.7	24.1	18.8	16.0	18.7	7.5	31.8	27.8	5.1	14.3	2.4
Iran	0.7		1.0	7.9	28.4	25.1	22.9	17.2	21.8	9.5	34.0	28.3	7.4	15.4	2.9
Indian	0.0	-1.0		6.3	25.1	22.4	16.5	14.5	17.2	7.1	29.8	26.5	6.2	15.0	2.5
Portugal	-5.8	-7.9	-6.3		19.2	18.8	11.3	9.3	12.1	1.1	28.3	23.9	-0.4	10.1	-0.5
Inner_Mongolia	-25.7	-28.4	-25.1	-19.2		3.8	-15.0	-12.7	-8.5	-20.5	15.1	13.8	-20.2	-3.2	-11.8
Liaoning	-24.1	-25.1	-22.4	-18.8	-3.8		-15.0	-13.2	-10.4	-18.0	10.9	11.3	-18.8	-5.4	-13.4
Xinjiang	-18.8	-22.9	-16.5	-11.3	15.0	15.0		0.3	4.4	-10.4	25.4	20.6	-10.8	5.3	-5.3
Altai	-16.0	-17.2	-14.5	-9.3	12.7	13.2	-0.3		3.2	-8.8	22.8	19.8	-9.6	4.8	-5.2
Chukotka	-18.7	-21.8	-17.2	-12.1	8.5	10.4	-4.4	-3.2		-11.9	20.6	18.0	-12.8	2.5	-6.6
Bryansk	-7.5	-9.5	-7.1	-1.1	20.5	18.0	10.4	8.8	11.9		27.8	24.2	-1.3	9.5	-1.0
Shanxi	-31.8	-34.0	-29.8	-28.3	-15.1	-10.9	-25.4	-22.8	-20.6	-27.8		1.7	-29.0	-14.3	-19.7
China_X	-27.8	-28.3	-26.5	-23.9	-13.8	-11.3	-20.6	-19.8	-18.0	-24.2	-1.7		-24.3	-14.1	-20.7
Iberia	-5.1	-7.4	-6.2	0.4	20.2	18.8	10.8	9.6	12.8	1.3	29.0	24.3		10.2	-0.3
Qinghai	-14.3	-15.4	-15.0	-10.1	3.2	5.4	-5.3	-4.8	-2.5	-9.5	14.3	14.1	-10.2		-12.4
Tibet	-2.4	-2.9	-2.5	0.5	11.8	13.4	5.3	5.2	6.6	1.0	19.7	20.7	0.3	12.4	

D(Andean_fox, W13_Guizhou; P3, P4)

X/Y	Ancient_Taimyr	Iran	Indian	Portugal	Inner_Mongolia	Liaoning	Xinjiang	Altai	Chukotka	Bryansk	Shanxi	China_X	Iberia	Qinghai	Tibet
Ancient_Taimyr		-0.8	-0.6	6.5	27.1	27.3	20.3	16.0	18.8	8.8	32.2	29.8	5.0	13.2	3.4
Iran	0.8		0.1	9.1	27.8	26.8	27.5	18.4	21.9	10.5	33.5	29.0	7.0	14.4	3.9
Indian	0.6	-0.1		7.2	24.9	23.7	20.2	16.0	18.7	8.4	31.0	27.7	5.6	14.3	4.0
Portugal	-6.5	-9.1	-7.2		19.1	20.3	12.3	9.5	11.9	1.3	26.4	24.5	-1.9	9.3	0.3
Inner_Mongolia	-27.1	-27.8	-24.9	-19.1		5.5	-13.5	-11.3	-7.1	-20.0	15.6	13.8	-21.4	-2.7	-10.3
Liaoning	-27.3	-26.8	-23.7	-20.3	-5.5		-15.1	-13.6	-11.4	-20.6	9.2	9.3	-22.1	-6.1	-12.2
Xinjiang	-20.3	-27.5	-20.2	-12.3	13.5	15.1		-0.8	3.8	-12.3	23.0	19.9	-15.1	4.1	-4.7
Altai	-16.0	-18.4	-16.0	-9.5	11.3	13.6	0.8		3.6	-8.7	21.8	19.5	-10.9	4.2	-4.3
Chukotka	-18.8	-21.9	-18.7	-11.9	7.1	11.4	-3.8	-3.6		-11.7	19.6	18.1	-14.3	1.9	-5.9
Bryansk	-8.8	-10.5	-8.4	-1.3	20.0	20.6	12.3	8.7	11.7		26.6	24.8	-3.3	9.1	-0.2
Shanxi	-32.2	-33.5	-31.0	-26.4	-15.6	-9.2	-23.0	-21.8	-19.6	-26.6		0.5	-29.7	-14.1	-18.9
China_X	-29.8	-29.0	-27.7	-24.5	-13.8	-9.3	-19.9	-19.5	-18.1	-24.8	-0.5		-25.8	-13.2	-18.3
Iberia	-5.0	-7.0	-5.6	1.9	21.4	22.1	15.1	10.9	14.3	3.3	29.7	25.8		10.6	1.2
Qinghai	-13.2	-14.4	-14.3	-9.3	2.7	6.1	-4.1	-4.2	-1.9	-9.1	14.1	13.2	-10.6		-10.0

Tibet	-3.4	-3.9	-4.0	-0.3	10.3	12.2	4.7	4.3	5.9	0.2	18.9	18.3	-1.2	10.0	

D(Andean_fox, W2_Jiangxi; P3, P4)

X/Y	Ancient_Taimyr	Iran	Indian	Portugal	Inner_Mongolia	Liaoning	Xinjiang	Altai	Chukotka	Bryansk	Shanxi	China_X	Iberia	Qinghai	Tibet
Ancient_Taimyr		0.3	1.4	3.0	23.3	24.9	18.5	14.9	16.0	5.1	28.5	24.7	3.9	26.0	27.0
Iran	-0.3		1.7	3.3	22.6	24.9	25.5	14.7	17.7	5.4	28.8	23.7	4.5	26.8	27.3
Indian	-1.4	-1.7		1.6	18.7	20.7	16.1	11.7	13.5	2.9	24.3	20.9	2.4	26.5	27.6
Portugal	-3.0	-3.3	-1.6		17.2	19.3	14.6	10.3	12.3	1.5	24.2	20.7	1.1	24.4	25.4
Inner_Mongolia	-23.3	-22.6	-18.7	-17.2		4.2	-9.5	-7.5	-6.6	-19.8	11.1	9.7	-16.0	18.2	21.1
Liaoning	-24.9	-24.9	-20.7	-19.3	-4.2		-13.3	-10.8	-10.7	-20.6	6.0	6.7	-17.9	16.4	19.8
Xinjiang	-18.5	-25.5	-16.1	-14.6	9.5	13.3		-0.2	1.4	-14.9	18.8	15.7	-12.9	21.8	23.5
Altai	-14.9	-14.7	-11.7	-10.3	7.5	10.8	0.2		1.2	-10.4	15.5	14.4	-9.3	21.0	23.1
Chukotka	-16.0	-17.7	-13.5	-12.3	6.6	10.7	-1.4	-1.2		-12.2	16.5	14.3	-10.7	20.1	22.2
Bryansk	-5.1	-5.4	-2.9	-1.5	19.8	20.6	14.9	10.4	12.2		26.0	22.2	-0.3	24.7	25.7
Shanxi	-28.5	-28.8	-24.3	-24.2	-11.1	-6.0	-18.8	-15.5	-16.5	-26.0		1.7	-23.1	13.1	17.1
China_X	-24.7	-23.7	-20.9	-20.7	-9.7	-6.7	-15.7	-14.4	-14.3	-22.2	-1.7		-19.4	11.9	16.1
Iberia	-3.9	-4.5	-2.4	-1.1	16.0	17.9	12.9	9.3	10.7	0.3	23.1	19.4		23.9	25.2
Qinghai	-26.0	-26.8	-26.5	-24.4	-18.2	-16.4	-21.8	-21.0	-20.1	-24.7	-13.1	-11.9	-23.9		12.4
Tibet	-27.0	-27.3	-27.6	-25.4	-21.1	-19.8	-23.5	-23.1	-22.2	-25.7	-17.1	-16.1	-25.2	-12.4	

D(Andean_fox, W6_Jilin; P3, P4)

X/Y	Ancient_Taimyr	Iran	Indian	Portugal	Inner_Mongolia	Liaoning	Xinjiang	Altai	Chukotka	Bryansk	Shanxi	China_X	Iberia	Qinghai	Tibet
Ancient_Taimyr		-1.7	-2.0	5.4	25.7	22.3	15.9	15.1	18.2	7.6	24.1	21.5	6.1	6.8	-1.2
Iran	1.7		-0.8	9.3	28.8	24.3	25.5	15.9	22.5	10.6	26.2	21.8	10.4	8.4	-0.3
Indian	2.0	0.8		7.6	24.0	22.0	18.3	14.0	19.4	8.8	22.6	20.7	8.9	8.6	-0.1
Portugal	-5.4	-9.3	-7.6		18.6	17.2	10.5	8.3	14.1	1.8	18.2	15.5	0.3	3.4	-4.1
Inner_Mongolia	-25.7	-28.8	-24.0	-18.6		3.9	-15.8	-10.0	-5.3	-19.0	3.0	2.4	-20.2	-8.9	-14.4
Liaoning	-22.3	-24.3	-22.0	-17.2	-3.9		-14.3	-11.2	-7.2	-16.9	-1.2	-1.1	-18.3	-10.9	-16.0
Xinjiang	-15.9	-25.5	-18.3	-10.5	15.8	14.3		1.9	7.2	-7.9	15.3	12.1	-11.6	-1.7	-8.7
Altai	-15.1	-15.9	-14.0	-8.3	10.0	11.2	-1.9		3.7	-8.1	11.8	9.2	-9.2	-2.5	-8.8
Chukotka	-18.2	-22.5	-19.4	-14.1	5.3	7.2	-7.2	-3.7		-12.0	6.9	5.9	-13.6	-5.2	-11.2
Bryansk	-7.6	-10.6	-8.8	-1.8	19.0	16.9	7.9	8.1	12.0		19.1	15.9	-1.7	2.1	-4.9
Shanxi	-24.1	-26.2	-22.6	-18.2	-3.0	1.2	-15.3	-11.8	-6.9	-19.1		0.0	-20.0	-10.6	-15.3
China_X	-21.5	-21.8	-20.7	-15.5	-2.4	1.1	-12.1	-9.2	-5.9	-15.9	0.0		-17.3	-10.3	-15.1
Iberia	-6.1	-10.4	-8.9	-0.3	20.2	18.3	11.6	9.2	13.6	1.7	20.0	17.3		3.2	-4.2
Qinghai	-6.8	-8.4	-8.6	-3.4	8.9	10.9	1.7	2.5	5.2	-2.1	10.6	10.3	-3.2		-10.1
Tibet	1.2	0.3	0.1	4.1	14.4	16.0	8.7	8.8	11.2	4.9	15.3	15.1	4.2	10.1	

D(Andean_fox, W7_Zhejiang; P3, P4)

X/Y	Ancient_Taimyr	Iran	Indian	Portugal	Inner_Mongolia	Liaoning	Xinjiang	Altai	Chukotka	Bryansk	Shanxi	China_X	Iberia	Qinghai	Tibet
Ancient_Taimyr		-1.0	-0.6	5.3	21.7	23.4	14.4	11.7	14.6	4.6	32.6	29.1	5.2	16.6	7.3
Iran	1.0		0.2	8.9	26.0	27.1	22.1	14.9	20.1	7.9	38.0	30.4	8.9	19.7	8.6
Indian	0.6	-0.2		7.1	21.5	22.9	16.0	12.6	15.8	5.8	31.7	28.2	7.0	18.9	8.5
Portugal	-5.3	-8.9	-7.1		15.3	17.6	8.1	6.3	9.8	-0.9	28.6	24.0	-0.4	13.2	4.4
Inner_Mongolia	-21.7	-26.0	-21.5	-15.3		4.7	-13.2	-11.8	-6.9	-19.0	16.6	14.9	-16.9	1.8	-4.4
Liaoning	-23.4	-27.1	-22.9	-17.6	-4.7		-16.5	-14.4	-10.6	-20.3	11.1	12.5	-19.6	-1.6	-6.8
Xinjiang	-14.4	-22.1	-16.0	-8.1	13.2	16.5		-0.5	4.3	-10.5	28.6	23.2	-9.0	9.7	1.1
Altai	-11.7	-14.9	-12.6	-6.3	11.8	14.4	0.5		3.7	-8.3	25.9	22.1	-6.8	9.0	1.2
Chukotka	-14.6	-20.1	-15.8	-9.8	6.9	10.6	-4.3	-3.7		-11.5	21.1	19.1	-10.2	6.1	-0.7
Bryansk	-4.6	-7.9	-5.8	0.9	19.0	20.3	10.5	8.3	11.5		31.2	26.5	0.6	13.8	4.8
Shanxi	-32.6	-38.0	-31.7	-28.6	-16.6	-11.1	-28.6	-25.9	-21.1	-31.2		2.6	-29.4	-9.8	-12.8
China_X	-29.1	-30.4	-28.2	-24.0	-14.9	-12.5	-23.2	-22.1	-19.1	-26.5	-2.6		-25.1	-10.7	-14.8
Iberia	-5.2	-8.9	-7.0	0.4	16.9	19.6	9.0	6.8	10.2	-0.6	29.4	25.1		13.3	4.6
Qinghai	-16.6	-19.7	-18.9	-13.2	-1.8	1.6	-9.7	-9.0	-6.1	-13.8	9.8	10.7	-13.3		-8.3
Tibet	-7.3	-8.6	-8.5	-4.4	4.4	6.8	-1.1	-1.2	0.7	-4.8	12.8	14.8	-4.6	8.3	

D(Andean_fox, W9_Heilongjiang; P3, P4)

X/Y	Ancient_Taimyr	Iran	Indian	Portugal	Inner_Mongolia	Liaoning	Xinjiang	Altai	Chukotka	Bryansk	Shanxi	China_X	Iberia	Qinghai	Tibet
Ancient_Taimyr		-1.4	-1.1	5.5	23.1	24.5	15.3	13.1	16.0	7.7	23.4	21.0	5.4	5.8	-2.4
Iran	1.4		0.0	9.1	25.4	25.6	25.7	14.7	20.5	9.3	24.4	21.8	9.3	7.8	-1.7
Indian	1.1	0.0		6.6	21.5	22.8	16.7	12.5	16.2	7.2	21.3	19.7	7.1	7.3	-1.7
Portugal	-5.5	-9.1	-6.6		15.9	19.4	9.7	7.5	12.2	1.6	16.1	14.7	-0.3	2.6	-5.1
Inner_Mongolia	-23.1	-25.4	-21.5	-15.9		7.1	-12.5	-9.4	-5.3	-16.6	2.3	3.0	-18.5	-9.8	-15.1
Liaoning	-24.5	-25.6	-22.8	-19.4	-7.1		-16.0	-14.0	-10.4	-19.8	-5.1	-3.8	-21.1	-13.2	-17.3
Xinjiang	-15.3	-25.7	-16.7	-9.7	12.5	16.0		0.8	6.1	-7.1	12.9	11.7	-11.3	-3.0	-9.8
Altai	-13.1	-14.7	-12.5	-7.5	9.4	14.0	-0.8		4.0	-7.5	11.4	10.2	-8.6	-2.9	-9.3
Chukotka	-16.0	-20.5	-16.2	-12.2	5.3	10.4	-6.1	-4.0		-10.2	7.0	6.9	-12.8	-5.8	-11.6
Bryansk	-7.7	-9.3	-7.2	-1.6	16.6	19.8	7.1	7.5	10.2		17.2	15.8	-1.8	1.3	-5.8
Shanxi	-23.4	-24.4	-21.3	-16.1	-2.3	5.1	-12.9	-11.4	-7.0	-17.2		1.0	-18.5	-10.8	-15.7
China_X	-21.0	-21.8	-19.7	-14.7	-3.0	3.8	-11.7	-10.2	-6.9	-15.8	-1.0		-17.1	-10.7	-15.6
Iberia	-5.4	-9.3	-7.1	0.3	18.5	21.1	11.3	8.6	12.8	1.8	18.5	17.1		2.6	-5.0
Qinghai	-5.8	-7.8	-7.3	-2.6	9.8	13.2	3.0	2.9	5.8	-1.3	10.8	10.7	-2.6		-10.5
Tibet	2.4	1.7	1.7	5.1	15.1	17.3	9.8	9.3	11.6	5.8	15.7	15.6	5.0	10.5	

Table S4. F3 tests among the SC, NA, and Tibetan gray wolves. Related to Figure 1 and Figure 2.

**Source 1**	**Source 1**	**Target**	**f_3**	**std. err**	**Z**	**SNPs**
Tibetan	SC	Jiangxi	-0.08023	0.007068	-11.352	2638661
Tibetan	NA	Jiangxi	-0.06734	0.007366	-9.141	2478583
Tibetan	SC	Qinghai	-0.09735	0.005096	-19.101	2631539
Tibetan	NA	Qinghai	-0.09298	0.00522	-17.811	2488007

Table S5. Z-scores for *D(Fox, X; Test, Y)*. Related to Figure 1 and Figure 2.

D(Andean_fox, P2; W12_Guizhou, P4)

X/Y	Ancient_Taimyr	Iran	Indian	Portugal	Inner_Mongolia	Liaoning	Xinjiang	Altai	Chukotka	Bryansk	Shanxi	China_X	Iberia	Qinghai	Tibet
Ancient_Taimyr		-5.7	-6.7	3.8	2.7	2.0	0.2	2.4	4.1	2.6	2.1	1.8	2.8	-8.5	-12.8
Iran	-4.1		38.2	14.5	4.4	2.5	14.5	11.5	8.0	14.9	1.5	1.5	14.8	-6.1	-10.8
Indian	-5.2	36.2		9.1	3.6	1.5	11.4	8.9	6.2	8.9	0.4	-0.2	8.6	-4.5	-8.7
Portugal	-1.8	8.2	4.4		4.6	2.9	11.9	8.3	8.3	19.8	1.0	-0.6	30.5	-7.4	-15.0
Inner_Mongolia	-22.4	-25.8	-21.6	-14.2		2.2	-9.6	-7.0	-3.4	-13.2	2.2	2.9	-17.6	-10.4	-14.7
Liaoning	-21.8	-24.4	-20.8	-16.7	-1.7		-12.7	-10.5	-7.0	-15.0	1.3	1.8	-16.5	-10.9	-15.3
Xinjiang	-18.2	-6.7	-6.0	1.2	7.5	3.7		8.1	4.8	1.3	3.0	2.7	-2.0	-8.0	-12.1
Altai	-11.7	-5.0	-4.6	0.7	5.0	2.1	6.3		5.0	3.1	2.3	3.4	0.4	-6.7	-11.7
Chukotka	-12.3	-11.3	-10.1	-2.2	5.2	4.6	0.1	1.8		-2.5	1.7	2.8	-4.5	-8.5	-13.2
Bryansk	-4.3	7.5	3.4	20.3	4.8	3.4	10.2	10.5	7.6		1.9	1.8	18.5	-7.8	-12.8
Shanxi	-29.9	-32.0	-29.5	-26.9	-13.4	-9.5	-22.9	-20.6	-19.7	-25.0		4.2	-28.4	-14.0	-18.7
China_X	-27.1	-28.8	-28.0	-24.8	-12.3	-9.7	-20.6	-18.0	-16.9	-23.1	2.3		-24.3	-13.8	-19.9
Iberia	-2.5	7.1	3.1	30.3	2.8	3.2	8.4	9.1	7.1	19.7	1.9	1.2		-7.9	-13.2
Qinghai	-23.7	-22.7	-18.7	-18.6	-6.1	-4.7	-14.4	-11.4	-11.9	-18.8	0.3	-0.8	-21.3		24.1
Tibet	-19.4	-18.7	-14.6	-17.9	-2.4	-1.8	-8.4	-8.0	-8.3	-17.1	2.0	0.7	-17.8	34.6	

D(Andean_fox, P2; W13_Guizhou, P4)

X/Y	Ancient_Taimyr	Iran	Indian	Portugal	Inner_Mongolia	Liaoning	Xinjiang	Altai	Chukotka	Bryansk	Shanxi	China_X	Iberia	Qinghai	Tibet
Ancient_Taimyr		-5.3	-5.7	3.7	2.7	2.1	0.3	2.3	3.5	2.5	1.9	1.8	2.5	-7.8	-12.4
Iran	-4.4		35.9	14.8	4.2	2.4	14.7	11.3	7.7	14.3	1.4	1.2	13.1	-6.4	-11.3
Indian	-4.9	34.8		9.8	4.1	2.0	12.5	9.2	6.7	9.0	1.0	0.4	8.0	-4.1	-8.8
Portugal	-1.8	8.2	4.2		4.3	2.8	11.4	8.0	8.1	20.2	0.8	-0.7	29.6	-7.9	-15.6
Inner_Mongolia	-21.9	-24.4	-18.8	-12.7		3.5	-7.7	-6.0	-2.2	-12.6	3.6	4.2	-15.1	-9.6	-14.3
Liaoning	-22.3	-24.7	-20.9	-17.5	-2.4		-12.8	-11.4	-8.0	-15.9	0.5	1.3	-17.4	-10.8	-15.1
Xinjiang	-16.8	-7.3	-6.3	0.3	5.6	2.5		6.9	3.7	0.4	1.6	1.6	-2.5	-8.8	-12.8
Altai	-10.7	-5.1	-4.2	0.8	5.0	2.2	6.4		5.1	3.2	2.4	3.4	0.4	-6.6	-11.6
Chukotka	-11.7	-11.6	-9.7	-2.5	4.6	4.0	-0.3	1.5		-2.8	1.3	2.5	-4.4	-8.8	-14.1
Bryansk	-4.2	7.1	2.9	21.2	4.9	3.2	9.9	10.6	7.3		1.7	1.4	18.2	-8.9	-14.2
Shanxi	-28.3	-30.6	-28.3	-24.9	-11.9	-8.6	-21.0	-19.3	-19.4	-23.9		4.2	-24.9	-13.4	-18.5
China_X	-27.5	-27.9	-26.5	-24.7	-10.8	-8.0	-19.7	-16.4	-16.1	-22.4	3.7		-23.3	-13.0	-18.4
Iberia	-1.7	7.9	3.6	31.0	3.8	4.2	9.7	9.8	7.4	20.1	2.8	2.0		-7.3	-13.7
Qinghai	-22.2	-21.0	-16.5	-17.1	-5.3	-4.2	-12.9	-10.5	-10.7	-18.1	0.7	-0.3	-18.1		23.7
Tibet	-19.9	-18.7	-14.6	-18.5	-3.9	-3.0	-8.8	-8.8	-9.2	-17.5	0.5	-0.7	-18.3	33.5	

D(Andean_fox, P2; W2_Jiangxi, P4)

X/Y	Ancient_Taimyr	Iran	Indian	Portugal	Inner_Mongolia	Liaoning	Xinjiang	Altai	Chukotka	Bryansk	Shanxi	China_X	Iberia	Qinghai	Tibet
Ancient_Taimyr		3.4	2.2	8.5	8.7	8.7	7.5	8.2	8.3	8.0	8.4	8.4	8.2	-0.5	-8.7
Iran	3.2		33.5	16.2	9.3	8.3	15.3	13.3	10.9	16.2	7.3	7.3	14.6	0.9	-6.8
Indian	1.2	31.5		12.5	8.2	7.0	14.1	11.9	10.0	11.6	6.2	5.5	10.8	1.6	-4.8
Portugal	6.1	14.1	11.2		11.1	10.3	17.1	13.5	14.8	23.4	8.8	8.0	33.6	0.9	-9.0
Inner_Mongolia	-6.2	-6.0	-5.1	-0.8		10.3	5.0	4.6	7.3	-0.3	10.5	11.2	-2.3	1.8	-8.2
Liaoning	-9.0	-9.6	-9.3	-4.5	7.4		0.5	0.3	3.8	-3.7	9.3	9.9	-4.3	-0.6	-9.6
Xinjiang	-3.2	3.9	3.8	8.2	11.7	10.0		12.2	10.5	8.1	9.7	10.0	6.1	1.3	-7.1
Altai	-1.3	3.2	3.3	6.6	9.8	8.0	11.2		9.7	8.2	8.6	9.4	6.3	1.0	-7.0
Chukotka	-1.2	0.2	0.3	5.9	12.0	12.0	9.0	9.1		5.7	9.8	10.2	4.3	1.1	-7.6
Bryansk	5.0	13.1	9.8	23.3	11.4	11.0	15.4	15.5	13.2		10.1	9.8	20.8	0.9	-8.0
Shanxi	-12.1	-12.9	-12.8	-8.9	3.0	4.9	-4.2	-3.5	-2.1	-8.1		14.5	-8.5	-0.5	-10.8
China_X	-12.0	-13.2	-13.2	-10.4	2.0	4.0	-5.0	-3.6	-2.4	-8.6	13.3		-8.9	-2.3	-12.0
Iberia	5.1	10.9	8.4	31.4	8.7	9.3	12.6	12.7	11.0	20.2	8.6	7.8		0.1	-9.4
Qinghai	-28.3	-28.3	-26.2	-25.1	-18.3	-18.0	-23.2	-21.8	-21.8	-25.5	-15.4	-15.4	-26.1		16.9
Tibet	-34.5	-33.2	-31.8	-33.7	-27.0	-26.9	-29.5	-29.3	-29.4	-32.4	-25.6	-26.7	-33.4	3.4	

D(Andean_fox, P2; W7_Zhejiang, P4)

X/Y	Ancient_Taimyr	Iran	Indian	Portugal	Inner_Mongolia	Liaoning	Xinjiang	Altai	Chukotka	Bryansk	Shanxi	China_X	Iberia	Qinghai	Tibet
Ancient_Taimyr		48.6	41.7	49.2	56.0	51.6	61.0	50.7	52.2	47.2	54.3	48.3	46.8	31.9	18.1
Iran	42.9		81.3	62.1	59.3	55.9	80.2	60.5	62.2	59.0	57.5	52.3	65.3	35.4	21.8
Indian	36.5	80.5		51.9	49.4	48.9	74.4	52.0	57.1	48.1	50.0	45.0	54.6	34.1	22.5
Portugal	38.5	57.4	45.5		48.3	48.4	73.2	49.5	57.1	54.9	48.8	43.4	58.9	28.7	17.2
Inner_Mongolia	28.8	37.5	32.1	39.2		57.4	62.1	47.8	52.4	38.1	60.4	56.9	37.1	36.3	18.3
Liaoning	23.3	27.3	22.7	30.3	44.5		43.3	34.7	42.5	28.9	49.7	46.2	29.7	30.2	14.9
Xinjiang	40.2	60.5	54.5	60.5	74.7	68.9		68.3	68.6	55.6	71.3	63.2	59.6	39.5	21.7
Altai	35.9	49.8	44.7	47.8	62.8	53.7	71.2		58.5	49.0	60.9	53.3	48.0	33.6	19.2
Chukotka	32.1	41.7	36.5	43.9	57.0	58.6	60.0	47.5		41.8	57.0	52.9	42.9	32.4	16.2
Bryansk	43.1	64.4	50.8	60.5	61.7	59.1	73.4	61.0	59.9		62.8	52.9	62.4	31.3	17.7
Shanxi	12.9	14.6	12.5	19.3	36.8	35.5	30.9	26.5	30.6	19.7		38.1	20.2	23.0	11.0
China_X	9.0	9.4	8.4	12.3	27.2	30.3	21.9	19.5	21.9	13.6	32.8		12.7	16.6	8.0
Iberia	41.3	63.0	52.6	60.9	54.8	55.0	71.6	57.6	55.6	61.8	57.7	46.8		31.1	16.9
Qinghai	15.1	19.5	17.0	18.6	35.2	35.0	38.4	27.0	32.3	21.3	41.7	34.8	21.3		44.3
Tibet	14.4	20.1	19.2	17.0	33.8	33.7	35.6	26.1	26.2	20.7	36.1	33.9	17.9	54.2	

D(Andean_fox, P2; W6_Jilin, P4)

X/Y	Ancient_Taimyr	Iran	Indian	Portugal	Inner_Mongolia	Liaoning	Xinjiang	Altai	Chukotka	Bryansk	Shanxi	China_X	Iberia	Qinghai	Tibet
Ancient_Taimyr		-6.1	-7.7	3.0	1.6	0.8	-1.0	1.5	2.8	2.0	1.0	0.9	2.2	-8.6	-13.3
Iran	-4.1		35.5	12.8	5.2	2.6	13.4	13.1	6.7	18.9	1.5	0.9	14.3	-5.3	-10.0
Indian	-4.8	31.9		8.1	6.1	3.0	11.2	12.0	6.2	12.8	1.6	0.5	9.0	-3.0	-7.8
Portugal	-2.6	6.1	2.8		4.8	2.5	9.1	8.8	7.3	23.1	0.2	-2.0	30.0	-6.7	-14.6
Inner_Mongolia	-25.2	-24.1	-19.8	-13.3		2.9	-8.8	-7.8	-3.2	-14.4	3.1	3.6	-17.0	-9.0	-13.8
Liaoning	-22.6	-24.2	-21.0	-15.6	-1.2		-12.4	-11.7	-6.3	-16.2	1.8	2.3	-16.5	-9.4	-14.9
Xinjiang	-18.7	-7.2	-6.5	1.2	7.1	3.6		8.3	4.5	1.5	2.7	2.2	-1.9	-8.0	-12.1
Altai	-13.1	-5.9	-5.3	-0.5	3.0	0.3	4.0		3.2	1.9	0.4	1.5	-0.9	-7.5	-12.6
Chukotka	-14.6	-13.3	-12.4	-4.1	2.4	1.5	-3.0	-0.7		-5.1	-1.3	-0.2	-6.5	-10.6	-15.3
Bryansk	-5.9	5.6	1.6	18.6	3.8	1.6	8.6	10.4	6.4		0.1	-0.4	17.5	-8.7	-14.0
Shanxi	-24.8	-26.0	-23.1	-18.1	0.0	3.3	-12.6	-12.0	-8.7	-19.1		16.1	-19.8	-4.0	-11.0
China_X	-22.2	-24.0	-22.3	-17.9	0.6	3.5	-12.4	-9.1	-6.6	-16.4	16.3		-17.8	-5.0	-11.7
Iberia	-4.2	6.0	1.7	31.3	1.5	2.0	7.8	9.2	5.9	20.4	0.3	-0.6		-9.1	-14.6
Qinghai	-21.0	-14.3	-10.6	-10.4	1.6	2.4	-6.2	-6.7	-4.8	-16.5	8.5	6.4	-14.1		27.1
Tibet	-18.3	-16.6	-11.3	-14.9	2.5	2.3	-4.0	-5.1	-5.2	-15.9	6.8	4.3	-15.9	36.9	

D(Andean_fox, P2; W9_Heilongjiang, P4)

X/Y	Ancient_Taimyr	Iran	Indian	Portugal	Inner_Mongolia	Liaoning	Xinjiang	Altai	Chukotka	Bryansk	Shanxi	China_X	Iberia	Qinghai	Tibet
Ancient_Taimyr		-5.1	-7.2	2.7	2.3	1.5	0.1	2.0	3.5	2.5	1.7	1.4	2.6	-7.9	-12.5
Iran	-3.7		35.6	12.9	5.0	2.8	14.0	12.5	7.0	19.5	1.4	0.6	15.2	-5.0	-9.8
Indian	-5.5	29.8		7.1	5.1	2.1	10.5	9.9	5.4	10.9	0.7	-0.3	8.2	-3.2	-7.8
Portugal	-2.9	6.2	2.8		4.7	2.4	9.5	8.6	7.4	24.5	-0.5	-2.5	29.9	-7.0	-14.8
Inner_Mongolia	-23.3	-21.5	-18.5	-11.4		4.0	-6.7	-6.6	-1.9	-13.0	4.2	4.7	-15.7	-8.1	-12.9
Liaoning	-23.2	-24.0	-22.2	-17.4	-3.6		-13.7	-13.6	-8.3	-17.8	-0.8	-0.3	-17.7	-11.3	-15.5
Xinjiang	-17.7	-6.5	-6.1	1.1	6.7	3.8		8.3	4.8	1.3	2.7	1.8	-1.6	-7.9	-11.7
Altai	-12.3	-5.1	-5.2	-0.2	4.0	1.0	5.4		4.0	2.2	1.2	2.1	-0.3	-6.6	-11.5
Chukotka	-12.8	-12.3	-11.2	-3.2	3.7	3.0	-1.5	0.3		-4.2	-0.2	1.1	-5.1	-9.5	-14.5
Bryansk	-5.8	5.5	1.6	18.5	3.9	1.9	8.5	9.8	6.2		-0.5	-0.8	17.5	-9.2	-14.2
Shanxi	-24.3	-24.2	-22.8	-17.6	1.5	4.9	-11.3	-9.8	-7.6	-18.8		17.0	-18.9	-3.1	-10.1
China_X	-21.9	-24.1	-22.2	-18.4	1.4	4.1	-12.1	-9.4	-6.2	-18.5	16.2		-18.5	-4.6	-11.2
Iberia	-3.2	7.3	2.4	31.1	2.2	2.9	9.1	8.8	6.2	21.5	0.7	-0.4		-8.9	-14.1
Qinghai	-19.2	-12.7	-9.5	-9.6	3.5	3.8	-4.8	-5.4	-3.9	-16.7	9.7	7.3	-12.9		27.7
Tibet	-15.8	-13.4	-9.5	-13.1	4.6	4.4	-1.1	-3.0	-3.1	-13.5	8.2	6.5	-14.5	37.2	

Table S6. D values and Z-scores for *D(Fox, Dhole/Jackal; Test, Jackal/Coyote/Red_wolf/Zhejiang)*. Related to Figure 1 and Figure 2.

**D(Fox, Jackal; P3, P4)**								
	**D-value**				**Z-score**			
**P3/P4**	**Dhole**	**Coyote**	**Red_Wolf**	**W7_Zhejiang**	**Dhole**	**Coyote**	**Red_Wolf**	**W7_Zhejiang**
**Dhole**		**0.624**	**0.619**	**0.527**		**100.0**	**100.0**	**100.0**
**Coyote**	**-0.624**		**0.017**	**-0.134**	**-100.0**		**6.4**	**-34.1**
**Red_Wolf**	**-0.619**	**-0.017**		**-0.147**	**-100.0**	**-6.4**		**-37.0**
**Ancient_Taimyr**	**-0.619**	**-0.042**	**-0.030**	**-0.189**	**-100.0**	**-16.2**	**-10.7**	**-39.7**
**W12_Guizhou**	**-0.614**	**-0.035**	**-0.022**	**-0.247**	**-100.0**	**-14.3**	**-8.9**	**-49.5**
**W13_Guizhou**	**-0.612**	**-0.033**	**-0.021**	**-0.205**	**-100.0**	**-13.4**	**-8.7**	**-41.8**
**W2_Jiangxi**	**-0.611**	**-0.033**	**-0.021**	**-0.194**	**-100.0**	**-12.9**	**-7.8**	**-42.7**
**W7_Zhejiang**	**-0.527**	**0.134**	**0.147**		**-100.0**	**34.1**	**37.0**	
**W6_Jilin**	**-0.612**	**-0.030**	**-0.019**	**-0.179**	**-100.0**	**-11.0**	**-6.4**	**-33.6**
**W9_Heilongjiang**	**-0.606**	**-0.030**	**-0.017**	**-0.170**	**-100.0**	**-10.5**	**-5.6**	**-29.9**
**Indian**	**-0.612**	**-0.037**	**-0.025**	**-0.179**	**-100.0**	**-12.8**	**-9.4**	**-43.5**
**Iran**	**-0.609**	**-0.035**	**-0.023**	**-0.175**	**-100.0**	**-14.3**	**-10.2**	**-50.6**
**Portugal**	**-0.611**	**-0.038**	**-0.026**	**-0.186**	**-100.0**	**-13.3**	**-9.9**	**-41.7**
**Inner_Mongolia**	**-0.610**	**-0.035**	**-0.023**	**-0.188**	**-100.0**	**-15.1**	**-9.5**	**-45.9**
**Liaoning**	**-0.612**	**-0.038**	**-0.026**	**-0.192**	**-100.0**	**-16.1**	**-10.7**	**-46.1**
**Xinjiang**	**-0.610**	**-0.036**	**-0.024**	**-0.186**	**-100.0**	**-16.5**	**-11.5**	**-55.5**
**Altai**	**-0.612**	**-0.038**	**-0.026**	**-0.184**	**-100.0**	**-15.3**	**-9.9**	**-44.3**
**Chukotka**	**-0.611**	**-0.036**	**-0.024**	**-0.186**	**-100.0**	**-14.5**	**-9.5**	**-45.5**
**Bryansk**	**-0.612**	**-0.039**	**-0.027**	**-0.181**	**-100.0**	**-15.4**	**-10.3**	**-42.0**
**Shanxi**	**-0.611**	**-0.037**	**-0.025**	**-0.191**	**-100.0**	**-16.4**	**-10.4**	**-45.0**
**China_X**	**-0.610**	**-0.038**	**-0.026**	**-0.195**	**-100.0**	**-15.0**	**-9.5**	**-40.5**
**Qinghai**	**-0.612**	**-0.037**	**-0.025**	**-0.193**	**-100.0**	**-13.3**	**-9.3**	**-43.1**
**Tibet**	**-0.610**	**-0.032**	**-0.019**	**-0.183**	**-100.0**	**-12.0**	**-6.9**	**-39.6**

**D(Fox, Dhole; P3, P4)**								
	**D-Value**				**Z-score**			
**P3/P4**	**Jackal**	**Coyote**	**Red_Wolf**	**W7_Zhejiang**	**Jackal**	**Coyote**	**Red_Wolf**	**W7_Zhejiang**
**Jackal**		**-0.004**	**-0.012**	**-0.117**		-1.5	**-3.9**	**-20.2**
**Coyote**	**0.004**		**-0.009**	**-0.114**	1.5		**-3.2**	**-19.2**
**Red_Wolf**	**0.012**	**0.009**		**-0.109**	**3.9**	**3.2**		**-17.8**
**Ancient_Taimyr**	**0.014**	**0.013**	**0.007**	**-0.109**	**4.6**	**4.1**	2.1	**-14.2**
**W12_Guizhou**	**0.020**	**0.017**	**0.011**	**-0.150**	**6.5**	**5.7**	**3.7**	**-19.4**
**W13_Guizhou**	**0.019**	**0.017**	**0.010**	**-0.125**	**5.9**	**5.4**	**3.2**	**-18.1**

**W2_Jiangxi**	**0.020**	**0.017**	**0.010**	**-0.119**	**6.6**	**5.6**	**3.4**	**-17.8**
**W7_Zhejiang**	**0.117**	**0.114**	**0.109**		**20.2**	**19.2**	**17.8**	
**W6_Jilin**	**0.028**	**0.024**	**0.017**	**-0.102**	**7.5**	**7.2**	**4.7**	**-11.7**
**W9_Heilongjiang**	**0.026**	**0.024**	**0.017**	**-0.108**	**7.0**	**6.3**	**4.2**	**-12.1**
**Indian**	**0.016**	**0.013**	**0.006**	**-0.113**	**5.6**	**4.7**	2.2	**-17.3**
**Iran**	**0.016**	**0.013**	**0.006**	**-0.108**	**6.3**	**5.4**	2.6	**-19.7**
**Portugal**	**0.015**	**0.012**	**0.005**	**-0.117**	**4.9**	**4.3**	1.8	**-18.0**
**Inner_Mongolia**	**0.015**	**0.012**	**0.005**	**-0.114**	**5.6**	**4.6**	2.1	**-19.0**
**Liaoning**	**0.015**	**0.013**	**0.005**	**-0.114**	**5.2**	**4.4**	1.9	**-19.0**
**Xinjiang**	**0.014**	**0.011**	**0.004**	**-0.116**	**5.4**	**4.6**	1.6	**-22.4**
**Altai**	**0.013**	**0.010**	**0.003**	**-0.113**	**4.4**	**3.5**	1.0	**-18.2**
**Chukotka**	**0.017**	**0.014**	**0.007**	**-0.117**	**5.6**	**4.9**	2.5	**-19.8**
**Bryansk**	**0.015**	**0.012**	**0.005**	**-0.114**	**5.0**	**3.9**	1.6	**-17.9**
**Shanxi**	**0.013**	**0.010**	**0.003**	**-0.124**	**4.4**	**3.6**	1.0	**-20.5**
**China_X**	**0.017**	**0.014**	**0.007**	**-0.115**	**5.5**	**4.8**	2.3	**-17.3**
**Qinghai**	**0.015**	**0.012**	**0.005**	**-0.117**	**5.2**	**4.5**	1.9	**-18.1**
**Tibet**	**0.017**	**0.015**	**0.007**	**-0.112**	**5.5**	**4.7**	2.5	**-17.0**

Transparent Methods

Detailed methods of this paper include the following:

- CONTACT FOR RESOURCE SHARING

- EXPERIMENTAL MODEL AND SUBJECT DETAILS

- METHOD DETAILS

- + Extraction

- + Library preparation

- + Sequencing and data processing

- + Genotype calling

- QUANTIFICATION AND STATISTICAL ANALYSIS

- + Phylogeny, maximum likelihood and neighbor joining

- + f3-statistics

- + D-statistics

- + TreeMix

- + F4-ratio test

- + Admixture Graph

- DATA AND SOFTWARE AVAILABILITY

- CONTACT FOR RESOURCE SHARING

Further information and requests for resources and reagents should be directed to and will be fulfilled by the Lead Contact Ya-Ping Zhang (zhangyp@mail.kiz.ac.cn).

- METHOD DETAILS

- + Extraction

Six historical wolf skin samples were collected from two museums: National Zoological Museum of China in Beijing, and Kunming Natural History Museum of Zoology in Yunnan. The Zhejiang wolf (W7_Zhejiang) was collected from Lin'an, Zhejiang province in 1974 and the Jiangxi wolf (W2_Jiangxi) was collected from Jiangxi province in May 1974. Both regions are located in the downstream region of the Yangtze River in South China. The two Guizhou wolves (W12_Guizhou and W13_Guizhou) were collected from Guizhou province in South China near Southeast Asia in 1963. The final two wolves sampled in this study (W9_Heilongjiang and W6_Jilin) were collected from Northeast China (Baoqing, Heilongjiang province on Jan 24th, 1957, and Baicheng, Jilin province on Feb 11th, 1957, respectively) (Figure 1A). All samples were treated by As2O3 for storage.

We extracted DNA from these six different skin samples. Each sample was shaved with a sterilized razor blade to remove the fur. For each skin sample, we cut 25 mg into small pieces of size <1 cubic millimeter, using sterilized scissors between each sample, placing the pieces into a PCR clean 2.0 mL DNA LoBind tube (Eppendorf, cat. No. 30108078). For each sample, we rinsed the pieces in 70% ethanol (Sigma Aldrich, cat. No. E7023). The mixture was vortexed at maximum speed for one minute and then spun at 13,200 rpm in a table centrifuge for one minute. Finally, we removed the resulting supernatant. We repeated these steps three times and let the tube stand for five minutes at 40°C for complete ethanol evaporation. We used the remaining skin sample in each tube to prepare 50 uL of DNA extract per sample, using the DNA extraction method described in Dabney et al (Dabney et al., 2013). Preparation of samples was performed in a clean room at the Laboratory on Molecular Paleontology, at the Institute of Vertebrate Paleontology and Paleoanthropology

(IVPP), Chinese Academy of Sciences, Beijing, China. All used tubes and other experiment materials were UV irradiated for 40 mins, and the used reagents were UV irradiated for 20 mins. All laboratory procedures were conducted using contamination controls, such as use of full body coverings, bleach decontamination, and UV irradiation of tools and work area before and between uses.

- + **Library preparation**

Thirty-five libraries were produced using a double stranded library preparation protocol (Kircher et al., 2012; Meyer and Kircher, 2010) (Table S1). Libraries were all treated with uracil-DNA-glycosylase (UDG) and endonuclease (Endo VIII) to remove characteristic ancient DNA deamination (Briggs et al., 2007). All 35 libraries were PCR amplified using AccuPrimePfx DNA polymerase (Life Technologies) (Dabney and Meyer, 2012). Sample-specific indexes were introduced into both the P5 and P7 adaptors during this library amplification to make it possible to distinguish samples from the new libraries from any other library (Kircher et al., 2012). Library concentrations were determined using a NanoDrop 2000 spectrophotometer and a DNA-1000 chip on the Agilent Bioanalyzer 2100.

- + **Sequencing and data processing**

We sequenced the libraries using 2×150 bp reads on an Illumina HiSeq Xten platform. Reads were demultiplexed according to the expected index pairs (Table S1) allowing one mismatch on each pair of reads. The resulting paired reads were then merged into a single read, requiring an overlap of at least 11 bp (with one mismatch allowed), using a modified form of SeqPrep (John, 2011), in which higher quality bases (and scores) are used in the overlap region. After stripping adapters, merged reads were aligned as unpaired molecules using BWA (v 0.6.1) using samse (Li and Durbin, 2009). Reads were considered duplicates if they had the same start and end positions, and all duplicates were removed with bam-rmdup (https://github.com/mpieva/biohazard-tools), keeping only the read for each set of duplicates with the highest quality bases (Table S1).

- + **Genotype calling**

We merged the SNPs from Wang et al (Wang et al., 2016) and Marsden et al (Marsden et al., 2016), excluding any variants where the alleles did not match and any SNPs only present in one of the datasets. Together, this merging yielded 13.74 million SNPs, including 4.25 million transversions. We used this SNP set to call alleles for other canids from previous studies (Auton et al., 2013; Botigué et al., 2017; Freedman et al., 2014; vonHoldt et al., 2016; Wang et al., 2019a; Wang et al., 2013; Wang et al., 2019b; Zhang et al., 2014), and for the samples in the present study. For all but the Jiangxi sample, we used random allele calling, choosing not to determine heterozygous sites, as the sequencing depths for most individuals are low (~0.15x-15.3x, Table 1). We applied a filter where we ignored the first and last two base pairs of each fragment, required a base pair quality higher than 20, a fragment length of no less than 30, and mapping quality of no less than 30. The W2_Jiangxi sample was sequenced to 37x (Table 1), a high enough coverage to call heterozygotes confidently. Thus, we applied the software GATK 3.3 with the Unified Genotyper parameter to determine diploid calls. For the two Guizhou samples (W12_Guizhou and W13_Guizhou), we also made diploid calls with GATK using a similar process as for W2_Jiangxi to test whether results were consistent or not. After comparing all the analyses, we found the test results are similar to those obtained using random calling without heterozygous sites.

- QUANTIFICATION AND STATISTICAL ANALYSIS

- + **Principal components analysis**

To investigate the relationship of the newly sampled individuals to wolf and dog populations, we calculated pairwise allele-sharing distances among all pairs of wolf and dog populations (Cavalli-Sforza, 1997). We applied a principal components analysis (PCA) to the resulting pairwise distance matrix using SMARTPCA (version: 13050) (Patterson et al., 2012). For the gray wolves (31 individuals, including six new individuals sequenced in this study from China), we grouped populations by region:

America, Europe, Middle East, North Asia, Tibet, and Southern China. In the PCA, the six new samples are presented with a black edge and are slightly larger than the points for other samples (Figure 1B). The PCA also includes other wolves and select domesticated dogs, including breed dogs from across the world and indigenous dogs from South and North China. The first PC distinguishes between gray wolf and dog populations, while the second PC distinguishes between East Asian and European dogs (Figure S2). We changed the following options in the default ***PAR*** file: altnormstyle: No; outliermode: 1 and 2 for Figure 1B and Figure S2, respectively.

- + Phylogeny

We constructed the phylogenetic relationship of 31 gray wolves (*Canis lupus*), one ancient wolf from Taimyr, two coyotes (*Canis latrans*), two jackals (*Canis aureus*), one red wolf (*Canis rufus*), one dhole (*Cuon alpinus*), and one Andean fox (*Lycalopex culpaeus*) using the MEGA-CC (Compute Core) for Linux systems (https://megasoftware.net/) (Kumar et al., 2016). The MEGA-Proto requires two setting files (.mao) – one is for generating a Maximum Likelihood phylogeny and the other is for generating a Neighbor-Joining phylogeny. Support values of each node were inferred using 1000 rapid bootstrap replicates, where all other settings are set to the defaults. The ***fasta*** file for the 39 individuals was converted from the EIGENSOFT format using a customized script, and degenerate base symbols were used to represent the heterozygotes.

- + f3-statistics

For f3 statistics, we use the same dataset as shown in Figure S2, but including the Jackal. We measured the shared genetic drift between each newly sequenced wolf (X) and other dogs, gray wolves, and the Jackal (Y). We computed statistics of the form *f3(X, Y; Dhole)* using qp3Pop (version: 412), which measures the shared genetic drift between populations X and Y since their separation from an outgroup (*Dhole*) (Raghavan et al., 2014). After ranking the f3 results, we drew a scatter plot with error bars for the six new samples (Figure S5).

- + D-statistics

We used *D*-statistics (Green et al., 2010; Meyer et al., 2012; Patterson et al., 2012) of the form *D(Fox, Test; X, Y)* and *D(Fox, X; Test, Y)* to formally test the relationship these samples have with different wolf populations using qpDstat (version: 712), where X and Y are 15 previously published wolves and Test are each of the six newly sequenced gray wolves. We divided the results into 12 sub-tables (Table S3 and S5), and the results were highlighted according to the magnitude of the Z values. We also used *D(Fox, Jackal/Dhole; Test, Jackal/Coyote/Red Wolf/Zhejiang)* to assess the ancient genetic component in the Zhejiang wolf (Table S6), where Test are each of the Dhole, Coyote, Red wolf, six newly sequenced wolves and 14 previously published wolves.

- + TreeMix

We applied TreeMix (v.1.13) (Pickrell and Pritchard, 2012) to investigate the relationship between the newly sequenced samples and wolf and dog populations. TreeMix determines population structure using maximum likelihood trees and allows for both population splits and potential gene flow by using genome-wide allele frequency data and a Gaussian approximation of genetic drift. To further investigate how well the tree model fits the data, we visualized the matrix of residuals for the tree model with no admixture. We test trees for zero, one, and two migration events (m). The maximum-likelihood tree for m = 0 (Figure S4) is based on 39 canids excluding dogs. Figure S6 and S7 are based on all canids and m = 0. Figure 2 and Figure S8 are based on all canids with m = 1 and m = 2, respectively. To assess how well supported the Treemix phylogeny is, each node was inferred using 1000 rapid bootstrap replicates, with all other settings set to default parameters. To visualize the tree and residual plot, we used the R script plotting_funcs.R, and the ***plot_tree*** and ***plot_resid*** functions, which are provided with the source code for Treemix (https://github.com/joepickrell/pophistory-tutorial/tree/master/example2).

- + F4-ratio test

In the D-statistic analyses, we observed that the Zhejiang wolf showed patterns indicating ancestry from a canid population that separated from wolves earlier than the dhole separated from wolves. To estimate the proportion of ancestry in the Zhejiang wolf that was contributed by admixture with a population more archaic than the dhole, we use the F4-ratio test, which provides an unbiased estimate of the admixture proportion (Reich et al., 2011). We used qpF4ratio (v1.0) from the ADMIXTOOLS software package (Patterson et al., 2012). We use the dhole and fox as the two source populations for the wolves (X), assuming an unrooted tree (Figure S9). Then, we expect the admixture proportion, α, in the f4-ratio test to be given by the following equation, where X is each gray wolf in turn.

$$\frac{f_4(Dhole,\ Jackal;\ Coyote,\ X)}{f_4(Dhole,\ Jackal;\ Coyote,\ Andean\ Fox)}$$

- + Admixture Graph

We used qpGraph (version: 6065) from the ADMIXTOOLS packages (Patterson et al., 2012) to test all possible relationships between the select individuals representing the major branches for canids. We began with a base graph using three samples (Andean_fox, Dhole and Ancient_Taimyr). Then we proceeded by attempting to fit the selected gray wolf samples (Gray_Wolf_Iberia, Gray_Wolf_Indian, W9_Heilongjiang, W7_Zhejiang and Gray_Wolf_Great_Lakes) in turn. If there are no models that fit, we change the order of addition and repeat the analysis.

We added Gray_Wolf_Iberia to all possible nodes of basal Admixture Graph either as a simple branch without mixture, or as a mixture between two branches. All together, we identified two models that fit the data (maximum |Z|<3, Figure S10). We then added Gray_Wolf_Indian to all possible nodes of these two graphs (Figure S10), either as a simple branch without mixture, or as a mixture between two branches. We

identified two models that fit the data (maximum |Z|<3, Figure S11). Adding W9_Heilongjiang to all possible nodes of the two graphs that fit the data for Gray_Wolf_Iberia and Gray_Wolf_Indian (Figure S11), we identified only one model that fit the data (maximum |Z|<3, Figure S12). We added W7_Zhejiang to all possible nodes of this graph (Figure S12) and identified one model that fit the data (maximum |Z|<3), Figure S13) We finally added Gray_Wolf_Great_Lakes to the previous graph (Figure S13) and identified only one model that fit the data (maximum |Z|<3, Figure S14).

We additionally tested adding other canids to the admixture graph, but no graph fit the data well (i.e. all maximum |Z|>3). From the final admixture graph (Figure S14), we confirmed patterns similar to that observed in other analyses and we estimated a similar admixture proportion (14%) for deep ancestry in W7_Zhejiang (Figure S14).

- DATA AND SOFTWARE AVAILABILITY

Sequence data for six gray wolf genomes has been submitted to the Genome Sequence Archive (http://gsa.big.ac.cn/) under accession number PRJCA001135. The SNP set will be made available on the iDog database (http://bigd.big.ac.cn/idog/).

References

Auton, A., Li, Y.R., Kidd, J., Oliveira, K., Nadel, J., Holloway, J.K., Hayward, J.J., Cohen, P.E., Greally, J.M., Wang, J., *et al.* (2013). Genetic Recombination Is Targeted towards Gene Promoter Regions in Dogs. Plos Genetics *9*.

Botigué, L.R., Song, S., Scheu, A., Gopalan, S., Pendleton, A.L., Oetjens, M., Taravella, A.M., Seregély, T., Zeeb-Lanz, A., Arbogast, R.-M., *et al.* (2017). Ancient European dog genomes reveal continuity since the Early Neolithic. Nature Communications *8*, 16082.

Briggs, A.W., Stenzel, U., Johnson, P.L., Green, R.E., Kelso, J., Prufer, K., Meyer, M., Krause, J., Ronan, M.T., Lachmann, M., *et al.* (2007). Patterns of damage in genomic DNA sequences from a Neandertal. Proc Natl Acad Sci U S A *104*, 14616-14621.

Cavalli-Sforza, L.L. (1997). Genetic and Cultural Diversity in Europe. Journal of Anthropological Research *53*, 383-404.

Dabney, J., Knapp, M., Glocke, I., Gansauge, M.T., Weihmann, A., Nickel, B., Valdiosera, C., Garcia, N., Paabo, S., Arsuaga, J.L., *et al.* (2013). Complete mitochondrial genome sequence of a Middle Pleistocene cave bear reconstructed from ultrashort DNA fragments. Proc Natl Acad Sci U S A *110*, 15758-15763.

Dabney, J., and Meyer, M. (2012). Length and GC-biases during sequencing library amplification: A comparison of various polymerase-buffer systems with ancient and modern DNA sequencing libraries. Biotechniques *52*, 87-+.

Freedman, A.H., Gronau, I., Schweizer, R.M., Ortega-Del Vecchyo, D., Han, E., Silva, P.M., Galaverni, M., Fan, Z., Marx, P., Lorente-Galdos, B., *et al.* (2014). Genome sequencing highlights the dynamic early history of dogs. PLoS Genet *10*, e1004016.

Green, R.E., Krause, J., Briggs, A.W., Maricic, T., Stenzel, U., Kircher, M., Patterson, N., Li, H., Zhai, W., Fritz, M.H., *et al.* (2010). A draft sequence of the Neandertal genome. Science *328*, 710-722.

John, J.S. (2011). SeqPrep (https://github.com/jstjohn/SeqPrep).

Kircher, M., Sawyer, S., and Meyer, M. (2012). Double indexing overcomes inaccuracies in multiplex sequencing on the Illumina platform. Nucleic Acids Res *40*, e3.

Kumar, S., Stecher, G., and Tamura, K. (2016). MEGA7: Molecular Evolutionary Genetics Analysis Version 7.0 for Bigger Datasets. Mol Biol Evol *33*, 1870-1874.

Li, H., and Durbin, R. (2009). Fast and accurate short read alignment with Burrows-Wheeler transform. Bioinformatics *25*, 1754-1760.

Marsden, C.D., Ortega-Del Vecchyo, D., O'Brien, D.P., Taylor, J.F., Ramirez, O., Vila, C., Marques-Bonet, T., Schnabel, R.D., Wayne, R.K., and Lohmueller, K.E. (2016). Bottlenecks and selective sweeps during domestication have increased deleterious genetic variation in dogs. P Natl Acad Sci USA *113*, 152-157.

Meyer, M., and Kircher, M. (2010). Illumina sequencing library preparation for highly multiplexed target capture and sequencing. Cold Spring Harb Protoc *2010*, pdb prot5448.

Meyer, M., Kircher, M., Gansauge, M.T., Li, H., Racimo, F., Mallick, S., Schraiber, J.G., Jay, F., Prufer, K., de Filippo, C.*, et al.* (2012). A High-Coverage Genome Sequence from an Archaic Denisovan Individual. Science.

Patterson, N., Moorjani, P., Luo, Y., Mallick, S., Rohland, N., Zhan, Y., Genschoreck, T., Webster, T., and Reich, D. (2012). Ancient admixture in human history. Genetics *192*, 1065-1093.

Pickrell, J.K., and Pritchard, J.K. (2012). Inference of population splits and mixtures from genome-wide allele frequency data. precedingsnaturecom http://precedings.nature.com/documents/6956/version/1.

Raghavan, M., Skoglund, P., Graf, K.E., Metspalu, M., Albrechtsen, A., Moltke, I., Rasmussen, S., Stafford, T.W., Jr., Orlando, L., Metspalu, E.*, et al.* (2014). Upper

Palaeolithic Siberian genome reveals dual ancestry of Native Americans. Nature *505*, 87-91.

Reich, D., Patterson, N., Kircher, M., Delfin, F., Nandineni, M.R., Pugach, I., Ko, A.M., Ko, Y.C., Jinam, T.A., Phipps, M.E.*, et al.* (2011). Denisova admixture and the first modern human dispersals into Southeast Asia and Oceania. Am J Hum Genet *89*, 516-528.

vonHoldt, B.M., Cahill, J.A., Fan, Z., Gronau, I., Robinson, J., Pollinger, J.P., Shapiro, B., Wall, J., and Wayne, R.K. (2016). Whole-genome sequence analysis shows that two endemic species of North American wolf are admixtures of the coyote and gray wolf. Sci Adv *2*, e1501714.

Wang, G.D., Shao, X.J., Bai, B., Wang, J.L., Wang, X.B., Cao, X., Liu, Y.H., Wang, X., Yin, T.T., Zhang, S.J.*, et al.* (2019a). Structural variation during dog domestication: insights from gray wolf and dhole genomes. National Science Review *6*, 110-122.

Wang, G.D., Zhai, W.W., Yang, H.C., Fan, R.X., Cao, X., Zhong, L., Wang, L., Liu, F., Wu, H., Cheng, L.G.*, et al.* (2013). The genomics of selection in dogs and the parallel evolution between dogs and humans. Nature Communications *4*, 1860-1860.

Wang, G.D., Zhai, W.W., Yang, H.C., Wang, L., Zhong, L., Liu, Y.H., Fan, R.X., Yin, T.T., Zhu, C.L., Poyarkov, A.D.*, et al.* (2016). Out of southern East Asia: the natural history of domestic dogs across the world. Cell Res *26*, 21-33.

Wang, X., Zhou, B.W., Yang, M.A., Yin, T.T., Chen, F.L., Ommeh, S.C., Esmailizadeh, A., Turner, M.M., Poyarkov, A.D., Savolainen, P.*, et al.* (2019b). Canine transmissible venereal tumor genome reveals ancient introgression from coyotes to pre-contact dogs

in North America. Cell Res.

Zhang, W., Fan, Z., Han, E., Hou, R., Zhang, L., Galaverni, M., Huang, J., Liu, H., Silva, P., Li, P., *et al.* (2014). Hypoxia adaptations in the grey wolf (*Canis lupus chanco*) from Qinghai-Tibet Plateau. PLoS Genet *10*, e1004466.
